# Epigenetic silencing of downstream genes mediated by tandem orientation in lung cancer

**DOI:** 10.1038/s41598-017-04248-w

**Published:** 2017-06-20

**Authors:** Steffen Kiehl, Tobias Zimmermann, Rajkumar Savai, Soni S. Pullamsetti, Werner Seeger, Marek Bartkuhn, Reinhard H. Dammann

**Affiliations:** 1 Institute for Genetics; Justus-Liebig-University, 35392 Giessen, Germany; 2 Department of Lung Development and Remodeling, Max-Planck-Institute for Heart and Lung Research, 61231 Bad Nauheim, Germany; 3 German Center for Lung Research (DZL), Universities of Giessen and Marburg Lung Center, 35392 Giessen, Germany

## Abstract

Epigenetic deregulation is of importance in tumorigenesis. In particular CpG islands (CGI), are frequently hypermethylated. Here, genome-wide DNA-methylation profiles of 480,000 CpGs in lung cancer cells were generated. It was observed that intra- and intergenic CGI exhibited higher methylation compared to normal cells. The functional annotation of hypermethylated CGI revealed that the hypermethylation was associated with homeobox domain genes and targets marked by repressive histone modifications. The strongest methylation variation was observed in transitional areas of CGI, termed shores. 5′-shores of promoter-associated CGI in lung cancer cell lines were higher methylated than 3′-shores. Within two tandem-oriented genes, a significant hypermethylation of the downstream-located CGI promoters was revealed. Hypermethylation correlates with the length of the intergenic region between such tandem genes. As the *RASSF1A* tumor suppressor gene represents such a downstream tandem gene, its silencing was analyzed using an inducible system. It was determined that the induction of an upstream gene led to a repression of RASSF1A through a process involving histone deacetylases and CPSF1. A tumor-specific increase in expression of histone deacetylases and *CPSF1* was detected in lung cancer. Our results suggest that the downstream gene could be susceptible to epigenetic silencing when organized in a tandem orientation.

## Introduction

Inactivation of tumor suppressor genes (TSG) is frequently observed in lung cancer and is accomplished through genetic and epigenetic mechanisms including a repressive chromatin state of the TSG promoter (e.g. aberrant DNA methylation)^1^. Hypermethylation of promoter-associated CpG islands (CGI) in particular represents a fundamental event in the epigenetic silencing of TSG during lung carcinogenesis^2^. CGI are sequences greater than 500 bp comprised of GC-rich and CpG dense elements in the genome notably, approximately 70% of known genes harbor CGI within their transcription start site^3^. The most frequently epigenetically inactivated TSG in lung cancer are the *Ras association domain family 1A* (*RASSF1A*) and the *cyclin-dependent kinase inhibitor 2A* (*CDKN2A*/*p16*) genes^4–7^.

Mechanisms, to maintain the inactive epigenetic state of CGI promoters are well studied. DNA methylation patterns are maintained by DNA methyltransferase 1 (DNMT1) and the 5-methyl-cytosines in turn serve as binding sites for the methyl-CpG binding domain (MBD) proteins^8^. These MBD proteins are directly involved in transcriptional repression by creating a compacted chromatin environment together with histone deacetylases (HDACs), histone methyltransferases (e.g. SUV39H1), and/or ATP-dependent chromatin remodeling machines^8, 9^. For example, inactive TSG promoter regions exhibit a repressed chromatin structure that lacks H3/H4 acetylation^1^. These repressive chromatin- and DNA-modifications maintain CGI-associated TSG promoters in an inactive heterochromatic state that is mitotically heritable and basically irreversible.

The initial trigger for the repressive modification of a TSG promoter and the exact dynamics of its epigenetic silencing are not yet understood in detail^10^. One hypothesis is that certain DNA sequences have a higher susceptibility to *de novo* methylation by DNMT3A and 3B^11^. Another hypothesis states that a bivalent chromatin pattern may predispose TSG to epigenetic silencing^12^. Furthermore, depending on the location, shielding against positive or negative regulatory effects from neighboring chromatin or transcription machinery may be required and hence insulator and boundary models have also been proposed^13–15^.

In order to investigate novel mechanisms that are involved in the epigenetic inactivation of TSG, we performed a global DNA methylation analysis in lung cancer cells. Here we report that for genes organized in a tandem orientation the downstream gene may be susceptible to epigenetic inactivation. This effect was correlated with the distance between the transcription end site of the upstream gene and the transcription start site of the downstream gene.

## Results

### Increased CpG methylation in lung cancer cells

**Figure 1:**
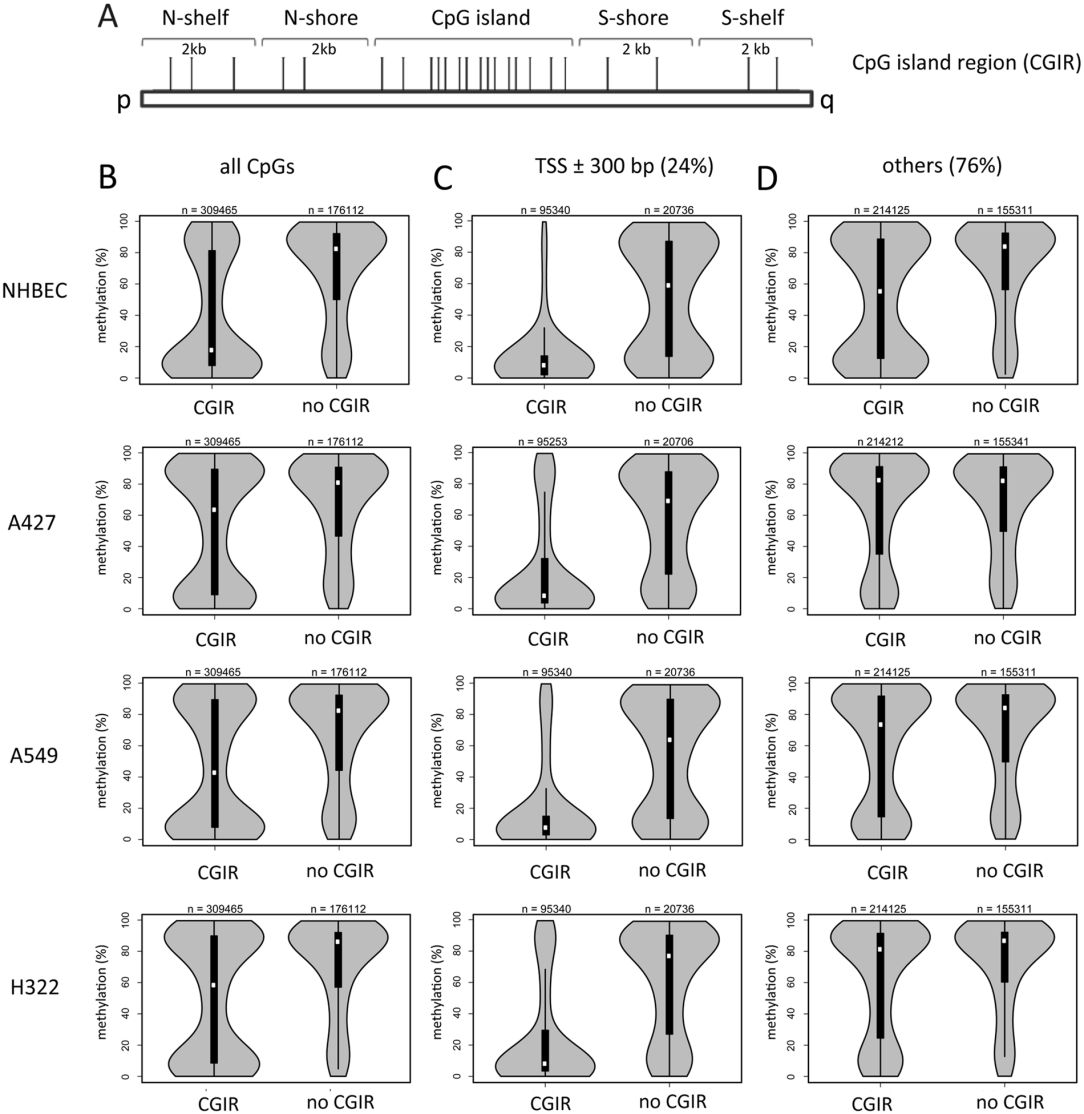
Increased methylation of CpG island-associated regions (CGIR) occurs in lung cancer cell lines. (**A**) GC and CpG rich genomic elements, termed CpG islands are flanked by 2 kb shore and 2 kb shelf regions. Depending on their orientation on the chromosome from the p- to q-arm these regions are denoted as N- or S-shores and shelves respectively. (**B**) Methylation levels of CpGs (n = 485,577) in CGIR (CpG island and flanking regions) and in no CGIR in normal human bronchial epithelial cells (NHBEC) and in three lung cancer cell lines (A427, A549 and H322) as quantified by 450 K bisulfite bead chip arrays and depicted in violin plots. (**C**) Methylation levels of transcription start site (TSS ± 300 bp flanking regions) associated CpGs (n = 116,076) were analyzed according to their location in CGI regions (CGIR) and no CGIR. (**D**) Methylation of CpGs outside of TSS (±300 bp) were analyzed according to the position in CGIR and no CGIR.

Epigenetic deregulation of tumor-associated genes is a frequent event in the pathology of lung cancer. However, distinct mechanisms that lead to aberrant DNA methylation are still under investigation. To dissect this further, we analyzed the DNA methylation patterns of over 480,000 CpG sites by bisulfite-based Illumina 450 K BeadChip arrays in normal human bronchial epithelial cells (NHBEC) and three non small cell lung cancer cell lines (NSCLC: A427, A549 and H322). CpGs on the array were annotated in CGI regions (CGIR), which consisted of a central CGI and 2 kb flanking shores and shelf regions (Fig. 1A). In NHBEC most CGIR-associated CpGs were rather weakly methylated (<20% methylation level) and only a few CGIR were strongly methylated (median methylation level 18%) (Fig. 1B). However, in the NSCLC a significant shift toward higher methylation levels of CGIR was observed (median methylation: 46%). For CpGs that were located outside of CGIR (no CGIR), this strong shift was not observed (Fig. 1B; median NHBEC: 82% and NSCLC: 83%). Genes associated with CGI that exhibited increased methylation in the lung cancer cell lines were subjected to a gene ontology (GO)-term analysis using different ontology data sets (Table S1). The GO-term analysis of hypermethylated genes revealed a significant enrichment of genes associated with homeobox domains and the function of sequence-specific DNA binding. Furthermore, the hypermethylated gene promoters were significantly enriched for genes that in ES cells harbor the repressive chromatin mark histone H3K27 trimethylation in their promoter sequences as well as for genes targeted by PRC2 and polycomb protein EED (Table S1).

We further classified CpG sites as those associated with a transcription start site (TSS ± 300 bp) and others (no TSS-associated CpGs) (Fig. 1C and D). TSS-associated CGIR were generally unmethylated in NHBEC (median 8.1%) and NSCLC (median 8.2%). CpGs associated with TSS, but not located at CGI exhibited high methylation levels in NHBEC (median: 72%) and in NSCLC (median: 59%) compared to CpGs located at TSS-associated CGIR. Conversely CGIR, which are not located proximal to a TSS exhibited tumor-specific hypermethylation was found (Fig. 1D). An increase of 26% in the methylation of NSCLC (median: 82%) compared to NHBEC (median 56%) was detected (Fig. 1D).

Previously, hypermethylation of promoter-associated CGI (e.g. *RASSF1A*) has been reported in lung cancers^2, 4^. Therefore, we identified genes that exhibited the highest methylation levels in A427, A549 and H322 compared to NHBEC (Table 1). Hypermethylation has already been reported for *PAX5, KCNAB3, MEIS2* and *GFRA3* in different cancer entities^16–20^. However, we also identified other genes that may represent novel epigenetically inactivated lung cancer-related target genes (e.g. *pyrimidinergic receptor P2RY﻿6 and PLK5*).

**Table 1:** Top 20 lung cancer-specific hypermethylated CGI-associated genes.

	CpG-islands	associated gene	CpG-island promoter methylation [%]					
			A427	A549	H322	Mean	NHBEC	methylation differences
1	chr11: 72975469–72975797	*P2RY6*	99	97	99	98	2	96
2	chr9: 37002489–37002957	*PAX5*	99	96	99	98	5	93
3	chr17: 7832532–7833164	*KCNAB3*	97	97	96	97	4	93
4	chr15: 37390175–37390380	*MEIS2*	96	97	94	96	4	92
5	chr5: 137610105–137610311	*GFRA3*	96	94	96	95	4	91
6	chr19: 1523705–1524565	*PLK5*	94	94	89	92	2	90
7	chr19: 18118741–18119553	*ARRDC2*	95	93	94	94	4	90
8	chr2: 228736230–228736544	*DAW1*	94	94	94	94	4	90
10	chr4: 682724–683079	*MFSD7*	93	94	94	94	4	90
9	chr4: 57371582–57372022	*ARL9*	95	94	95	95	5	89
11	chr14: 24837872–24838324	*NFATC4*	96	94	95	95	6	89
12	chr2: 27665251–27665670	*KRTCAP3*	96	96	97	96	7	89
13	chr17: 41177336–41177593	*RND2*	93	87	97	92	3	89
14	chr10: 102495116–102495613	*PAX2*	95	95	92	94	5	89
15	chr2: 54086776–54087266	*ASB3, GPR75*	92	93	93	93	4	89
16	chr12: 49487963–49488202	*DHH*	97	80	98	92	3	89
17	chr20: 44540445–44540957	*PLTP*	94	93	80	89	1	88
19	chrX: 100546063–100546550	*TAF7L*	96	96	96	96	8	86
18	chr15: 72489478–72490119	*GRAMD2*	93	93	94	93	6	87
20	chr12: 124246524–124247254	*DNAH10*	95	84	95	92	5	87
126	chr3: 50377803–50378540	*RASSF1A*	91	93	91	92	13	79

To examine the aberrant methylation of CGIR in further detail, we analyzed the methylation level of the CGI and flanking shore and shelf regions in 69 NSCLC, 6 normal lung cell lines (NLC) and 5 normal lung tissues (NLT) (Fig. S1). The methylation profiles of these samples have been published previously^21, 22^. The methylation level of NSCLC was significantly higher in the shores compared to CGI. Furthermore, in NSCLC a significantly increased methylation of shores (median: 55%) compared to normal cells (median 35%; p = 2.2 × 10^−16^) was revealed (Fig. S1). This 20% tumor specific increase in methylation was not observed for CGI (NSCLC: median 15% and NLC: 11%) or and shelf regions (NSCLC: 77% and NLC: 73%).

### Asymmetry in N- vs. S-shore methylation

**Figure 2:**
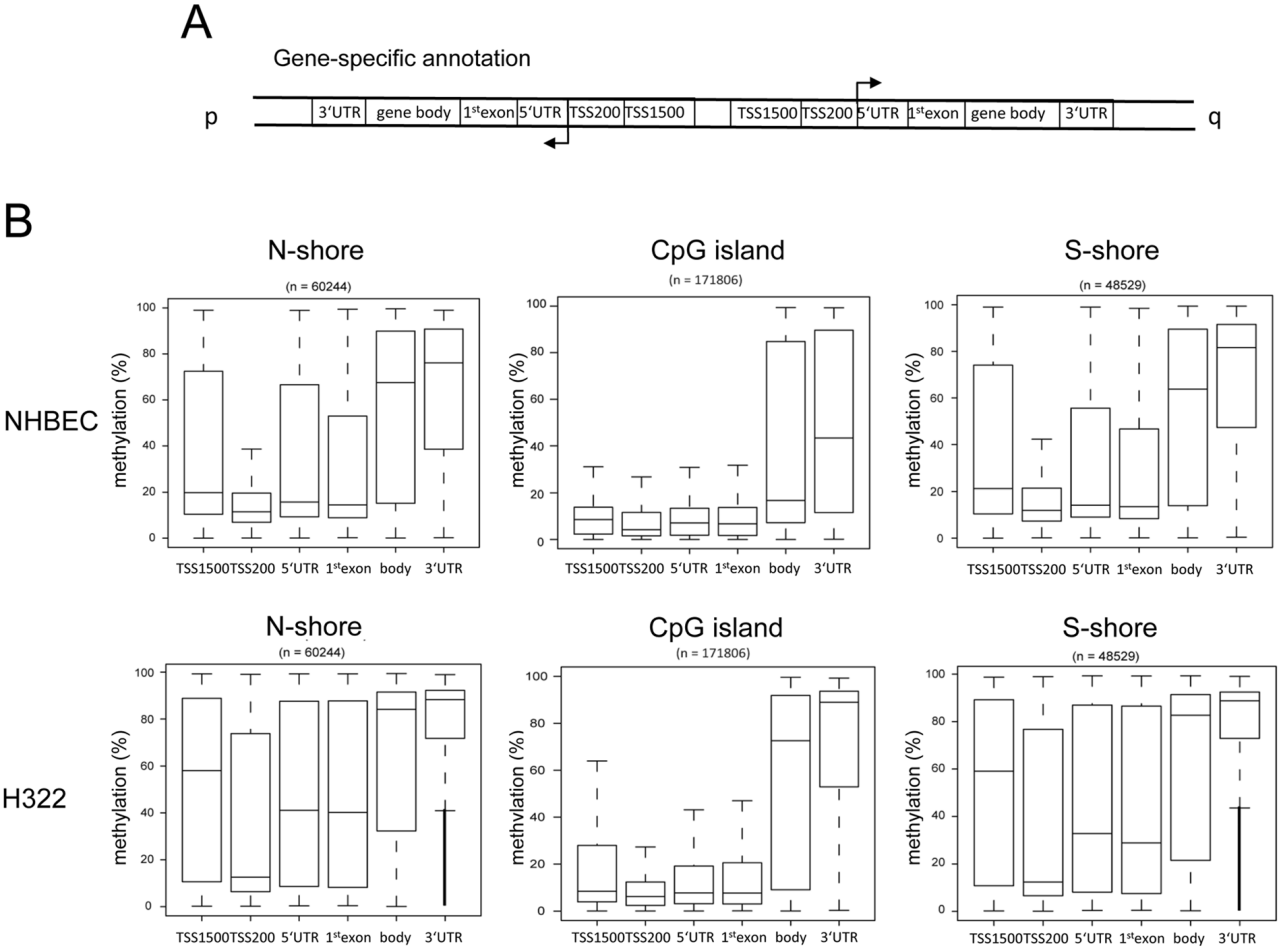
Methylation of gene specific CpGs in lung cancer cell lines. (**A**) CpG sites are annotated depending on the gene specific orientation in TSS1500 (−1500 to −200 bp), TSS200 (−200 bp to TSS), 5′-UTR, 1^st^ exon, gene body and 3′-UTR. (**B**) Methylation levels of CpG islands and flanking N-and S-shores were analyzed in NHBEC and in the lung cancer cell line H322 by 450 K bisulfite bead chip arrays according to the gene specific annotation.

**Figure 3:**
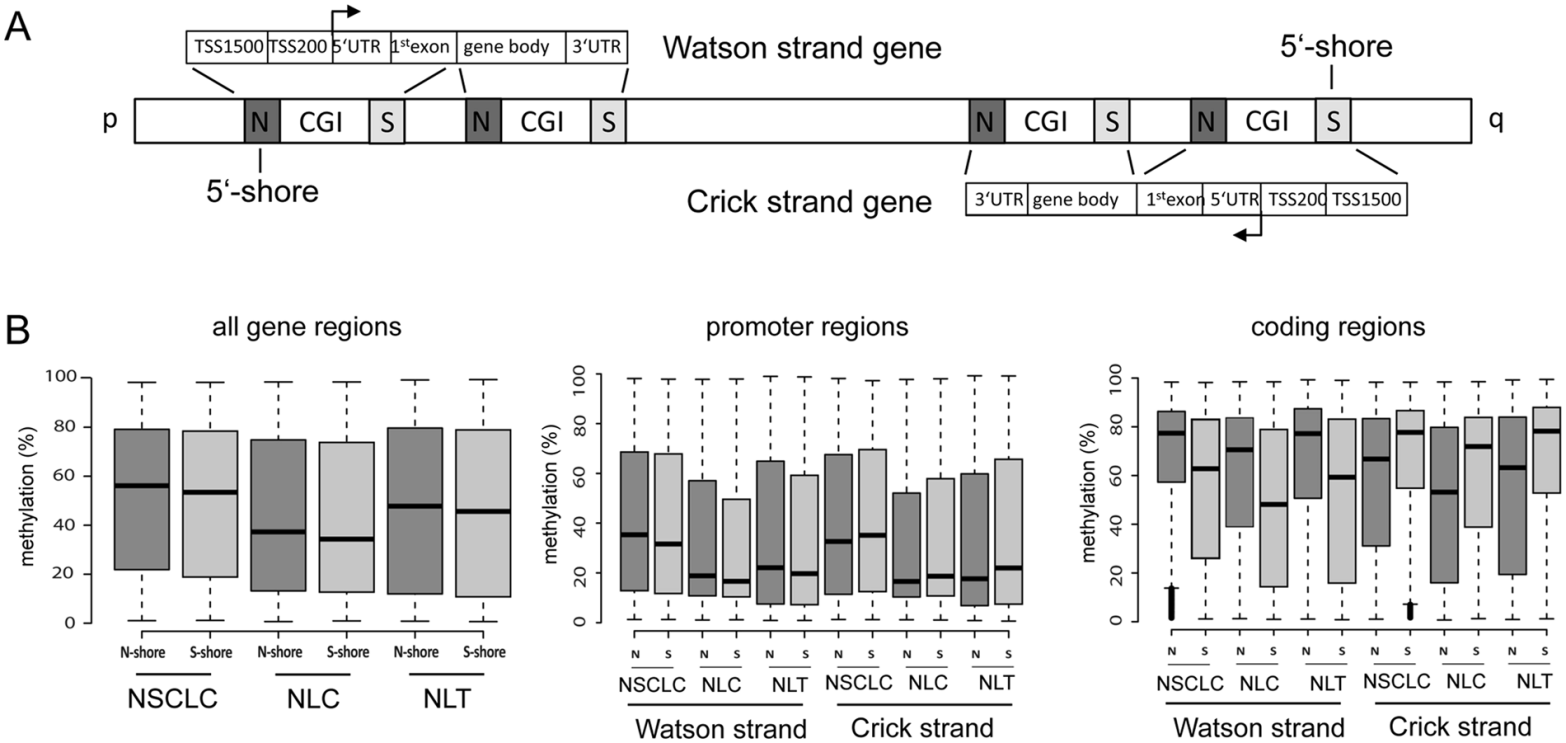
Increased 5′-shore methylation in promoter and coding regions. (**A**) Depending on the chromosome orientation genes are transcribed from the p- to the q arm (Watson strand genes) or in the opposite direction (Crick strand genes). For Watson strand genes the CGI flanking N-shores are the 5′-shores and for Crick strand genes S-shores are the 5′-shores. For further details are provided in Fig. 2. (**B**) Methylation levels of N- and S-shore gene specific regions in 69 NSCLC cell lines (ABC-1, SW1573, HOP18, HCC1171, LXFL529, H1703, H441, EBC-1, H322, A427, H1568, CHA-GO-K-1, H2087, H1975, H226, H2444, HCC515, H1299, H2030, H2405, HCC2935, H1373, H1666, RERF-LC-Ad1, Calu-6, H1155, H1651, H2347, H358, H838, H2228, HCC827, H2073, H1650, H23, H1993, Calu-3, H650, H460, H727, A549, H292, HCC4006, H2170, H1838, H820, H1355, RERF-LC-MS, H2122, H1793, H661, HOP62, HCC15, EKVX, H1792, H2110.1, Calu-1, H2110, HCC4017, H2009, HOP92, SK-MES-1, RERF-LC-KJ, H1437, H647, H2126, H2172, H1435 and H1755), six normal lung cell lines (NLC): gBEC1, gBEC1_UI, gSAC1, gSAC1_UI, gBEC and gSAC) and five normal lung tissues (NLT): GSM1264690, GSM1264711, GSM1264727, GSM1264764, GSM1264854). Methylation data were obtained from NLCBI-GEO-Accession: GSE36216 and GSE52401^21, 22^. Promoter regions are defined as the TSS1500 (−1500 to −200), TSS200 (−200 to TSS), 5′-UTR, 1^st^ exon and coding regions consisted of the gene body and 3′-UTR.

To dissect the mechanism of aberrant promoter methylation, we analyzed the methylation level of CGI and flanking shore regions in further detail (Figs 2 and 3). In the utilized array, shores were annotated according to their chromosome orientation from the p- to q-arms as in N- and S-shores, respectively (Fig. 1A). Additionally, 92% of CpG sites (n = 447,186) were annotated depending on the gene specific orientation in TSS1500 (−1500 to −200), TSS200 (−200 to TSS), 5′-UTR, 1^st^ exon, gene body and 3′-UTR (Fig. 2A). Initially, we analyzed whether the methylation levels in NHBEC and H322 were dependent on this gene specific annotation (Fig. 2B). In general, the methylation levels of the CGI at TSS1500, TSS200, 5′-UTR and 1^st^ exon were low (median <10%). In contrast, CGI located in gene bodies exhibited a higher methylation level in NHBEC (median 17%) and an additional tumor-specific increase in methylation was observed in H322 (median 72%). This increased methylation was also detected for the 3′-UTR in H322 (Fig. 2B). Furthermore for the N- and S-shore, we observed significantly increased methylation for all gene-specific regions in H322 compared to NHBEC.

Notably, the methylation levels of flanking shores different among all analyzed lung cells (Figs 2 and 3). In lung cancer cell lines but also in normal lung, the methylation levels were significantly higher in N-shores than in S-shores, (Fig. 3B). To further elucidate the differential methylation of the N- and S-shores, we analyzed the methylation of the shores according to their gene context. For this purpose, we grouped genes transcribed from p- to q-arm direction as Watson strand genes and those genes transcribed from the opposite strand as Crick strand genes (Fig. 3A). The N- and S-shores represent the 5′-shore and for the Watson and Crick strand genes, respectively. In NSCLC we observed that promoter-associated 5′-shores (TSS1500, TSS200, 5′-UTR and 1^st^ exon) exhibited a significantly higher methylation compared to 3′-shores (Fig. 3B). In normal lung the degree of promoter shore methylation was lower; however a similar tendency was revealed (Fig. 3B). We also analyzed the methylation of coding region-associated shores (gene body and 3′-UTR). As displayed in Fig. 2, we observed a high methylation of these regions in NSCLC but also normal lung samples. Additionally, we also found that the methylation level of 5′-shores is higher for the coding regions compared to the 3′-shores (Fig. 3B). To verify this finding for a specific gene, we analyzed the methylation of the *RASSF1A* CGI and flanking regions by bisulfite pyrosequencing (Fig. S2). Within the epigenetically inactivated NSCLC A427, A549 and H322, the *RASSF1A* TSS and its flanking regions were highly methylated. Notably, for the cell lines that harbored unmethylated TSS sites and expressed *RASSF1A*, the 5′-shore regions of the *RASSF1A* promoter exhibited higher methylation compared to the analyzed 3′-flanking region (Fig. S2).

### Epigenetic silencing of downstream genes located in tandem orientation

**Figure 4:**
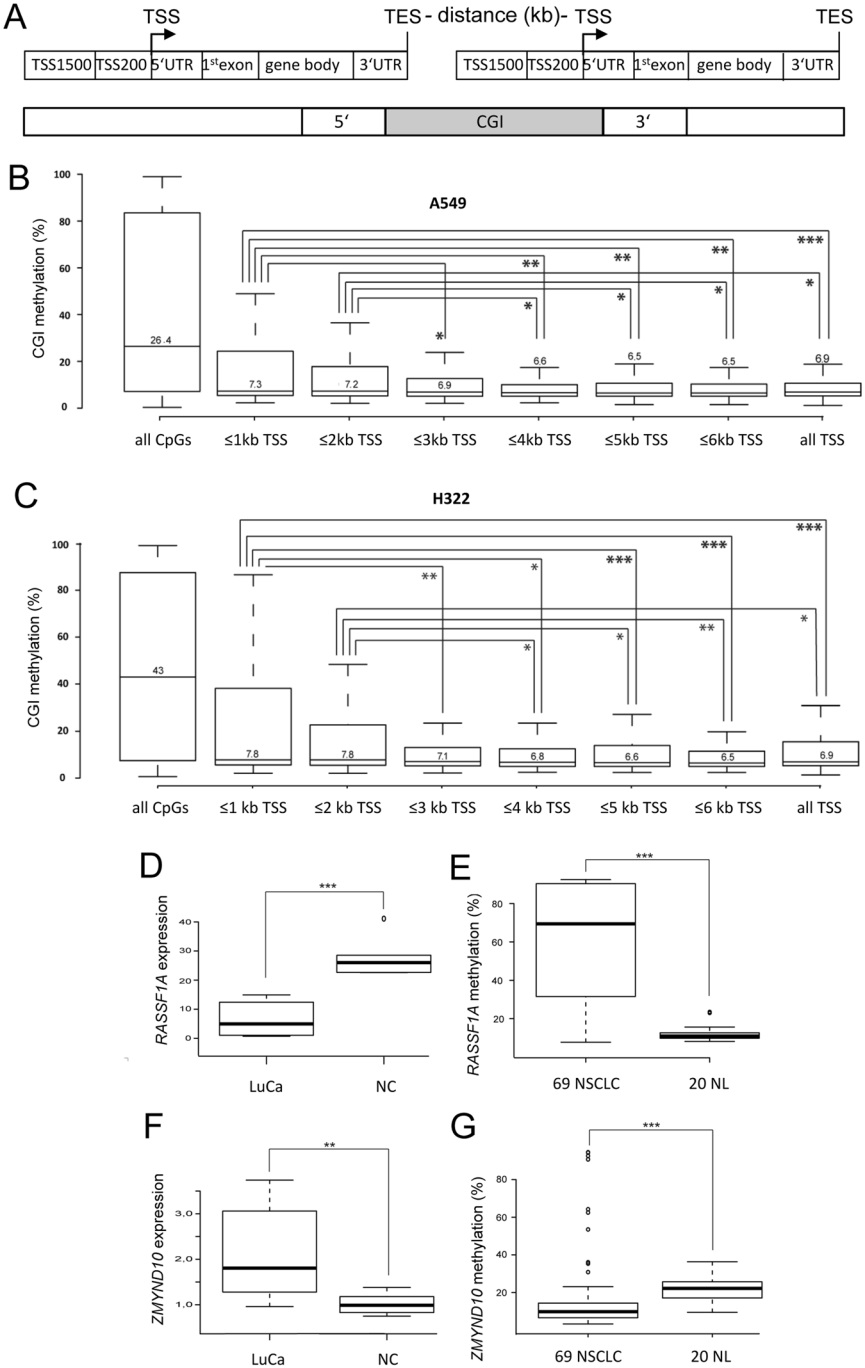
Epigenetic silencing of downstream genes located in tandem orientation. (**A**) Outline of the organization of tandem oriented genes with a CpG island at the transcriptional end site (TES) of the upstream gene and the transcriptional start site (TSS) of the downstream gene. (**B**) Methylation level of TSS-associated CGI in A549 cells was plotted depending on the distance between TES and TSS in kb (n = 258 to 297 CGI). Methylation as detected using 450 K bisulfite bead chip arrays. “All CpGs” and “all TSS” indicate the methylation level of CpGs located at CGI and TSS-associated CGI, respectively. (**C**) Methylation levels of tandem oriented downstream CGI in H322 cells. (**D**) *RASSF1A* expression levels were analyzed in lung cancer cell lines (LuCa: A427, A459, H322 and H358) and normal lung cells (NC: PAF and PASMC) by qRT-PCR. *RASSF1A* expression was normalized to the *GAPDH* level. (**E**) Methylation of *RASSF1A* was analyzed in 69 non small cell lung cancer (NSCLC) cell lines and 20 normal lung tissues (NL). For details see also Fig. 3. (**F**) *ZMYND10* expression levels were analyzed in LuCa and NC as measured by qRT-PCR and normalized to *GAPDH* levels. (**G**) Methylation of *ZMYND10* was analyzed in 69 NSCLC cell lines and 20 NL. *p < 0.05, **p < 0.01 and ***p < 0.001.

Next, we were interested in analyzing the methylation level of CGI promoters that are located proximal to the termination end site (TES) of an upstream gene (Fig. 4). Thus we analyzed the methylation level of CGI-associated with the transcriptional start site (TSS) at 1, 2, 3, 4, 5 and 6 kb from the TES and TSS (Fig. 4A–C). Initially, CGI methylation was compared to all TSS-associated CGIs in A549 and H322 cells (Fig. 4B and C) which demonstrated that in general, TSS-associated CGIs exhibited low levels of methylation.

Notably, we observed that TSS located ≤2 kb downstream of TES displayed a significantly increased methylation compared to that at TSS located more than 4 kb downstream of TES (Fig. 4B and C). This data suggested that CGI promoters in close proximity to a TES exhibited increased methylation. Next, we annotated the 20 top downstream genes that showed a lung cancer specific hypermethylation in A549, H322 and A427 compared to normal lung tissues (Table 2). In particular, the tumor suppressor genes *RASSF1A* and *CDKN1C*, which have previously been described as epigenetically inactivated, were found among the identified genes located in this type of configuration^2, 4, 23–25^. The top annotated genes (Table 2) also include *AOX1* and *CPT1C*, which have previously been found to be hypermethylated in cancer as well^26, 27^. To verify the epigenetic inactivation of *RASSF1A* in lung cancer, we analyzed its CGI methylation and expression (Fig. 4D and E). *RASSF1A* expression in lung cancer was significantly downregulated compared to that in normal cells (Fig. 4D). Consequently the promoter methylation of *RASSF1A* was significantly higher in the 69 analyzed NSCLC vs. the to 20 normal lung samples analyzed (Fig. 4E). To understand the mechanism underlying this aberrant methylation, we examined the epigenetic status of the upstream-located *ZMYND10* gene (Fig. 4F and G), for which an inverse level of expression and methylation was observed in the analyzed samples. Specifically, in lung cancer the *ZMYND10* expression was elevated (Fig. 4F) and its promoter exhibited reduced methylation compared to normal tissue samples (Fig. 4G). These results suggested that the epigenetic silencing of *RASSF1A* may be associated with an activation of the upstream tandem gene *ZMYND10*.

**Table 2:** Top 20 tandem-oriented hypermethylated downstream genes.

	downstream CpG-island	downstream gene	Downstream CpG island methylation (%)							
			A549	H322	A427	mean LuCa	NLu1	NLu2	mean normal	methylation differences
1	chr2:201450526–201451027	*AOX1*	82	94	94	90	6	5	6	85
2	chr3:50377803–50378540	*RASSF1A*	93	91	91	92	7	8	8	84
3	chr14:24803678–24804353	*ADCY4*	90	93	92	92	12	7	10	82
4	chr17:40932329–40933299	*WNK4*	92	94	93	93	15	15	15	78
5	chr19:50193020–50194798	*CPT1C*	92	90	92	91	14	13	14	77
6	chr3:49314437–49314815	*C3orf62*	53	95	96	81	5	5	5	77
7	chr11:64066757–64068741	*TEX40*	81	79	85	82	12	11	12	70
8	chr17:72931729–72932601	*OTOP30*	78	90	88	86	19	16	17	68
9	chr11:66045211–66045708	*CNIH2*	28	97	86	70	3	4	3	67
10	chr1:36771830–36773009	*SH3D21*	37	93	92	74	8	8	8	66
11	chr1:21043832–21044771	*KIF17*	60	75	66	67	8	9	9	59
12	chr17:72855621–72858012	*GRIN2C*	35	48	93	59	8	8	8	51
13	chr17:6679205–6679710	*FBXO39*	23	88	87	66	20	14	17	49
14	chr12:7023261–7024089	*ENO2*	38	54	92	61	13	13	13	48
15	chr11:64509433–64513826	*RASGRP2*	21	69	59	49	6	6	6	43
16	chr17:46670522–46671458	*PHPT1*	23	51	72	49	7	6	6	43
17	chr2:74729399–74731166	*LBX2*	59	36	86	60	19	17	18	42
18	chr6:30881533–30882296	*VARS2*	23	58	67	49	10	9	10	40
19	chr11:62368454–62370491	*MTA2*	53	37	55	48	12	20	16	33
20	chr11:2907308–2907675	*CDKN1C*	51	38	51	47	17	15	16	31

### Activation of *ZMYND10* results in inactivation of the *RASSF1A* promoter

**Figure 5:**
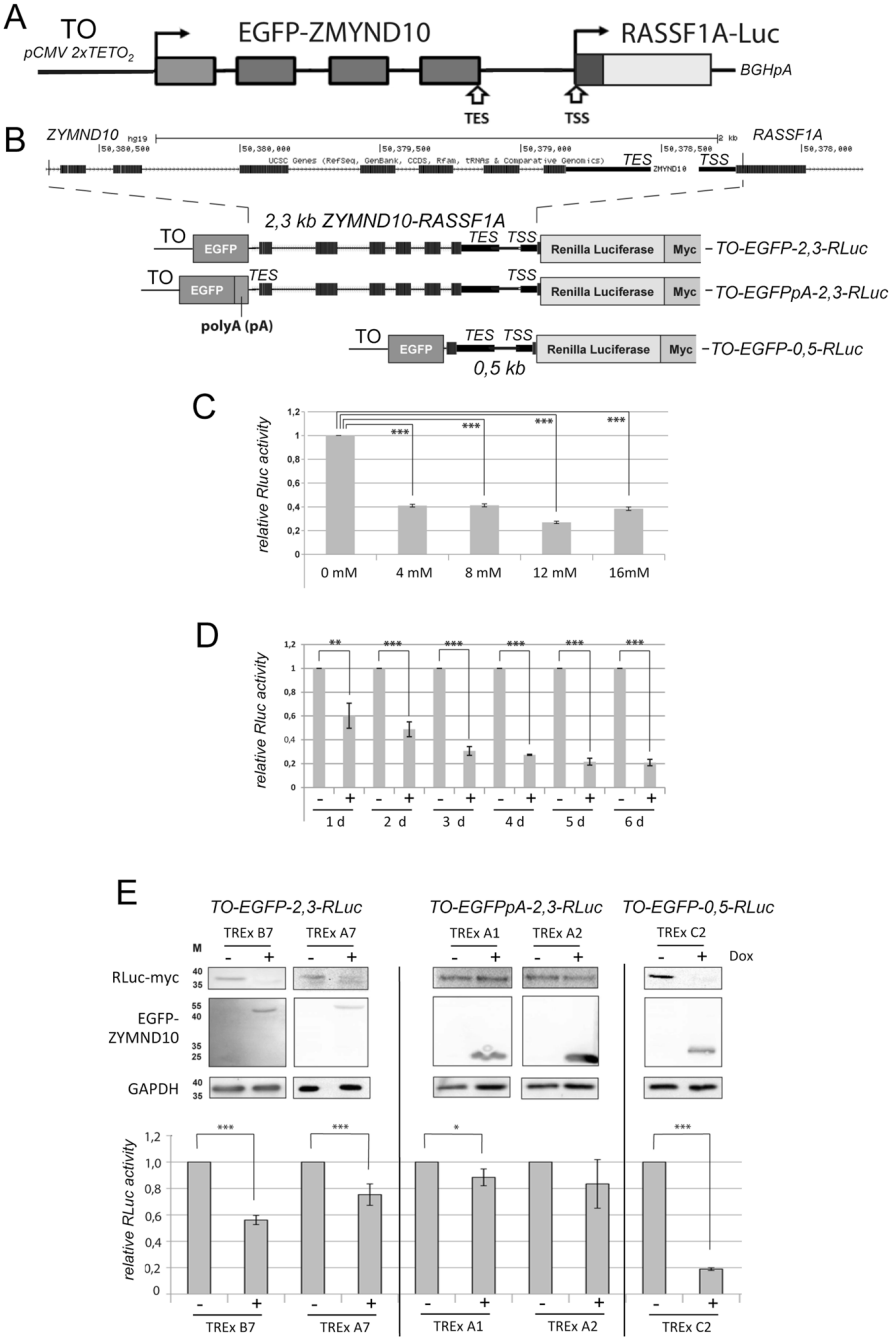
Induction of an upstream gene correlates with the downregulation of a tandem oriented downstream promoter. (**A**) Outline of the tandem oriented reporter gene construct. The *ZMYND10* - *RASSF1A* promoter including the *ZYMND10* transcriptional end site (TES) and the *RASSF1A* promoter and transcriptional start site were cloned in a tetracycline inducible vector system (Tet -On/TO). The *EGFP*-*ZYMND* fusion gene was under the control of the pCMV 2xTETO_2_ promoter and the 500 bp *RASSF1A* promoter including 5′-UTR and 17 bp of Exon1α was ligated in frame to Myc tagged Renilla luciferase (RLuc). (**B**) UCSC genome browser view of the 2.3. kb *ZMYND10* - *RASSF1A* promoter locus. Black boxes represent exons. Outline of the genetic structure of the tandem reporter TO-EGFP-2,3-RLuc. In the construct TO-EGFPpA-2,3-RLuc a 300 bp SV40 poly A site (pA) was inserted at the 3′-end of *EGFP*. To generate the tandem reporter TO-EGFP-0,5-RLuc a 1.8 kb fragment of ZYMND10 was deleted. (**C**) Luciferase assay of the TO-EGFP-2,3-RLuc construct. The tandem reporter was stably transfected into TREx293 cells and induced for four days with the indicated concentration of doxycycline (Dox). RLuc activity was analyzed as described in the methods section. (**D**) Time dependent downregulation of RLuc activity. TREx293 cells stably transfected with TO-EGFP-2,3-RLuc were induced with 4 mM Dox (+) or uninduced (−) for the indicated days (d) and RLuc activity was analyzed. (**E**) Analysis of different tandem constructs by western blot and luciferase assay. Two TREx clones (B7 and A7) of the stable transfected TO-EGFP-2,3-RLuc were induced with 4 mM Dox for four days and the protein levels were subsequently analyzed by western blot. Protein lysates were separated on SDS PAGE and blotted. Full-length blots are included in the supplementary information file. For detection of the indicated proteins primary antibody against myc, GFP and GAPDH were utilized. In parallel RLuc activity was analyzed with a luciferase assay. RLuc luciferase activity was normalized to transient transfected firefly luciferase. Additionally the expression of RLuc and EGFP were analyzed in two TO-EGFPpA-2,3-RLuc TREx clones (A1 and A2) and the C2 clone of TO-EGFP-0,5-RLuc by western blot and luciferase assay. *p < 0.05, **p < 0.01 and ***p < 0.001.

To dissect the mechanism involved in the epigenetic silencing of a tandem oriented downstream promoter, we cloned a 2.3 kb fragment of the *ZMYND10-RASSF1A* locus that included the TES of *ZMYND10* and the full length *RASSF1A* promoter in an inducible reporter system (Fig. 5). The TES of *ZYMND10* and the TSS of *RASSF1A* are in close proximity (170 bp). The obtained construct (*TO-EGFP-2,3-RLuc*) allowed for the inducible expression of a GFP-ZMYND10 fusion protein under the control of a tetracycline/doxycycline (Dox) regulated Tet-On (TO) promoter (Fig. 5A and B). *TO-EGFP-2,3-RLuc* was stably transfected in TREx-293 cells, which continuously express the tetracycline repressor. In this system, the activity of the downstream *RASSF1A* promoter was analyzed by a luciferase (RLuc) assay (Fig. 5B and C). After 4 days of Dox treatment with different concentrations (4 to 16 mM) we observed the decrease in the RLuc activity under the control of the *RASSF1A* promoter (Fig. 5C). Additionally, we observed a decreased expression of RLuc in a time-dependent manner from 1 day (40% reduction) to 6 days (80% reduction) with 4 mM Dox treatment (Fig. 5D). Subsequently, we generated two TREx clones (B7 and A7) and analyzed the expression of these tandem genes by western blot and luciferase assay (Fig. 5E). Both clones expressed the *EGFP-ZYMND10* fusion protein after Dox treatment (Fig. 5E). RLuc expression was reduced after induction of the upstream gene at the protein level along with a significant reduction of luciferase activity (Fig. 5E) inducting of the upstream located TO promoter induced occlusion of the downstream *RASSF1A* promoter.

To analyze this mechanism in detail, we cloned the SV40 polyA (pA) site proximal to *EGFP* in order to generate a novel TES 2 kb upstream of the *RASSF1A* TSS (Fig. 5B). The expression of this construct termed TO-EGFPpA-2,3-RLuc was analyzed in two different TREx clones (A1 and A2) by western blot and luciferase assay (Fig. 5E). Under these conditions we observed Dox induced expression of *EGFP*, but only a weak reduction in RLuc activity and expression (Fig. 5E). Interestingly, this effect was significantly less pronounced compared to the original constructs that contained a distance of 170 bp between the TES and TSS. Thereafter, we deleted 1.8 kb of the coding region of *ZYMND10*, which resulted in a close proximity (0.5 kb) between the TO promoter and the *RASSF1A* promoters (Fig. 5B). This construct TO-EGFP-0,5-RLuc was analyzed without and with Dox induction of the upstream promoter (Fig. 5E). Here, we also found a drastic downregulation of RLuc expression on protein and activity levels. These results indicating that the occlusion of the *RASSF1A* promoter correlated with the distance to the upstream promoter and the TES.

### Epigenetic deregulation of promoter sequences in lung cancer

**Figure 6:**
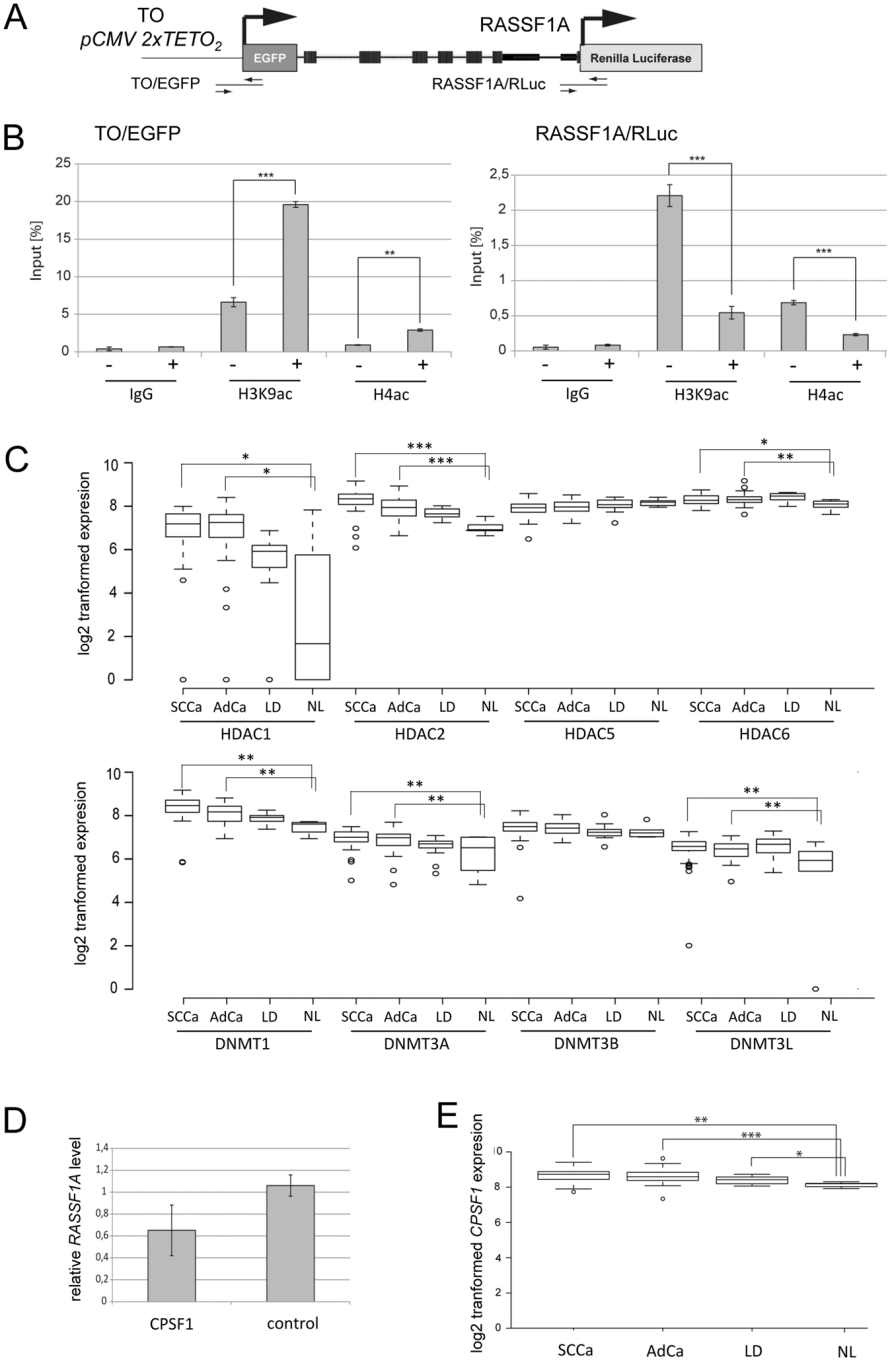
Epigenetic regulation of tandem genes in lung cancer. (**A**) Outline of the tandem oriented reporter gene construct analyzed by chromatin immuno-precipitation (ChIP). The analyzed TO/EGFP and RASSF1A/RLuc promoter regions are depicted. For details see Fig. 5. (**B**) TREx293 cells stably transfected with TO-EGFP-2.3-RLuc (clone B7) were induced with 4 mM Dox (+) or uninduced (−) for four days (d) and histone modifications were analyzed by ChIP. Chromatin was precipitated with anti histone H3K9ac, histone H4ac and IgG antibodies (negative control) antibodies. DNA was isolated and analyzed by qPCR. Levels are plotted relative to 1% of input sample. (**C**) Expression of *HDAC1*, *HDAC2*, *HDAC5*, *HDAC6*, *DNMT1*, *DNMT3A*, *DNMT3B* and *DNMT3L* was analyzed by microarray in 49 primary squamous cell lung cancer (SCCa), 41 primary lung adenocarcinoma (AdCa), 15 non malignant lung disease samples (LD) and 6 normal lung tissues (NL)^2^. Data are depicted as log2 transformed expression. (**D**) CPSF1 induced repression of RASSF1A. CPSF1 was cloned in pCMV-Tag1 and overexpressed in A427 cells. Endogenous *RASSF1A* level were analyzed by qRT-PCR and plotted relative to the empty control vector (=1). (**E**) Expression levels of *CPSF1* in SCCa, AdCa, LD and NL. *p < 0.05, **p < 0.01 and ***p < 0.001.

We next analyzed the epigenetic inactivation of the RLuc promoter by ChIP (Fig. 6). As histone acetylation (ac) represents the key epigenetic hallmark promoter activity, we analyzed the histone H3K9ac and H4ac at the TO/EGFP and RASSF1A promoters with and without induction of the upstream promoter in the tandem reporter assay by ChIP (Fig. 6A and B). Following Dox induction of the TO promoter, significantly increased acetylation of histone H3 and H4 were detected within this region. In contrast, at the *RASSF1A* promoter a significant reduction in histone H3K9ac and H4ac was found (Fig. 6B). The deactetylation of these histones was inactive of the epigenetic inactivation of the downstream promoter. To identify factors involved in this effect, we overexpressed different histone deacetylases (HDAC1/2/3/4/5/6/8/10 or Sirt1) and analyzed the expression of the RLuc promoter using the luciferase assay (Fig. S3). We found that overexpression of all analyzed histone deacetylases significantly repressed the activity of the *RASSF1A* promoter (Fig. S3).

To further dissect the epigenetic deregulation in primary lung cancer, we analyzed the expression levels of *HDAC1*, *HDAC2*, *HDAC5*, *HDAC6*, *DNMT1*, *DNMT3A*, *DNMT3B* and *DNMT3L* in 49 SCCa, 41 AdCa, 15 not malignant lung disease samples (LD) and 6 normal lung tissues (NL) (Fig. 6C). Expression levels were obtained a microarray previously generated in our laboratory^2^. Compared to normal lung samples, we observed significantly higher levels of *HDAC1*, *HDAC2*, *HDAC6*, *DNMT1*, *DNMT3A* and *DNMT3L* in primary SCCa and AdCa (Fig. 6C). Significantly increased levels of *UHRF1BP*, *c-Myc* and *EZH2* were also observed in cancer samples compared to normal lung (Fig. S4). However, for *HDAC5* and *DNMT3B* this increase was not significant (Fig. 6C).

To reveal additional factors that might be involved in the epigenetic occlusion of the downstream gene by HDACs and the transcriptional machinery, the published interactome data of the histone deacetylase family was analyzed^28^. In particular, the physical interaction of HDAC6 with cleavage and polyadenylation specificity factor 1 (CPSF1) has been reported^28^. CPSF1 is involved in the regulation of transcription termination by RNA polymerase II. Accordingly, we analyzed the effect of *CPSF1* overexpression on endogenous *RASSF1A* expression (Fig. 6D), which demonstrated reduced *RASSF1A* levels in the *CPSF1* transfected cells (Fig. 6D). In addition, we compared *CPSF1* levels in primary lung SCCa, AdCa and normal lung (Fig. 6E), from which a significant increase of *CPSF1* expression was detected in the lung cancer samples compared to normal lung. These results suggested that *CPSF1* could be involved in the epigenetic inactivation of *RASSF1A* in lung cancer.

## Discussion

The epigenetic inactivation of TSG by promoter hypermethylation represents a key event in the pathogenesis of lung cancer. Depending on the genetic and environmental context several mechanisms have been reported that may contribute to the silencing of genes in cancer. Deregulation of epigenetic key factors, including DNMT, HDAC and nucleosome remodelers and modifiers have been reported in lung cancer and may represent interesting therapeutic targets^29–34^. In the present study, we revealed that for genes localized in tandem orientation the downstream gene could be susceptible to epigenetic promoter occlusion depending on the distance between the TES of the upstream gene and the TSS of the downstream gene (Fig. 4). Notably, the *RASSF1A* lung tumor suppressor gene is located in such a configuration, suggesting that its promoter occlusion might contribute to its epigenetic silencing which constitutes a frequent event in lung and other cancers^2, 4, 35^. Here, we confirmed that *RASSF1A* is frequently hypermethylated in 69 NSCLC; conversely the upstream gene *ZMYND10* is relatively hypomethylated in these tumor samples compared to normal lung tissues (Fig. 4E and G). To analyze the mechanism of *RASSF1A* occlusion in further detail and we generated an inducible tandem oriented reporter system that mimics the genomic organization of *RASSF1A* and its upstream gene *ZMYND10* (Fig. 5). We observed that induction of the upstream gene resulted in a decreased expression of the *RASSF1A* reporter gene. Furthermore, the effect was dependent on the distance between the polyA site and the transcriptional start site (TSS) of *RASSF1A* (Fig. 5). In addition, we also observed a distance dependency of the TSS-associated CGI hypermethylation as detected by the 450K array (Fig. 4B and C). Consistent with these observations, epigenetic inactivation of other genes, such as *CDKN1C*, *AOX1* or *CPT1C* that are located in a similar configuration, has also been reported in cancers^25–27^. It will be interesting to confirm the mechanism of downstream promoter occlusion in other tandem-oriented genes.

At the level of chromatin, we observed that the reduction of the *RASSF1A* promoter activity was associated with the deacetylation of histones H3 and H4 (Fig. 6). In lung cancer samples, we also observed a significant overexpression of *HDAC1*, *HDAC2*, *HDAC6* and also *DNMT1* and *DNMT3A* (Fig. 6). In comparison^36^, observed an increased expression of *HDAC6* in lung AdCa and the aberrant expression correlated negatively with patient prognosis. Recently, it has also been reported that HDAC6 suppresses *RASSF1A* expression^37^. The aberrant expression of *DNMT1* and *DNMT3* has also been documented for lung cancer^30, 38^. In particular, wild type TP53 regulates the *DNMT1* level and overexpression of DNMT1 has been correlated with mutated *p53* ^39^. The interaction of HDACs with DNMTs and CPSF1 has also been revealed^28, 40, 41^. As the upregulation of *CPSF4* in cancer has been reported^42^, we analyzed the expression of *CPSF1* and found that it was overexpressed in lung cancer (Fig. 6) and that its, overexpression resulted in reduced *RASSF1A* levels (Fig. 6D). Our data therefore suggest that the aberrant expression of *CPSF1*, *HDAC6* and DNMT in cancer may cause promoter occlusion of *RASSF1A*. This is consistent with results from our previous studies, which indicate that histone deacetylation precedes the hypermethylation of the *RASSF1A* promoter^43^.

Conversely, it is also important to note that DNMT and HDAC inhibitors reactivate *RASSF1A* expression in cancer^15, 43, 44^. It will be interesting to analyze if suppression of *ZMYND10* leads to reactivation of the *RASSF1A* in cancer cells. Since RNA interference reduces RNA levels by a post-transcriptional mechanism this technique is not useful to reduce *ZMYND10* transcription rate. However genomic editing with the CRISPR/Cas9 technology could be utilized to alter regulatory sequences of the *ZYMND10* gene. Another factor that might be involved in the reactivation of the *RASSF1A* tumor suppressor gene is the insulator binding protein CTCF^14, 15^. Specifically, it has been reported that CTCF separates chromatin boundaries and activates the expression of tumor suppressor genes by epigenetic mechanisms^13, 14, 45^.

The results from the present study also indicated that intra- and intergenic CGI are methylated to a higher degree in lung cancer cells than in normal lung. Furthermore, CGI regions located at the 3′-UTR exhibited a significant tumor-specific hypermethylation in lung cancer (Fig. 2) indicating that transcriptional termination may correlate with aberrant methylation of CGI at TES. This suggests that TES-associated CGI that are co-localized at TSS (e.g. the *RASSF1A* promoter) might be susceptible to epigenetic silencing. Additionally, we observed a correlation between the direction of transcription (5′-3′) and the methylation of CGI. 5′-shores of promoter-associated CGI in lung cancer cell lines were significantly more highly methylated than 3′-shores. We verified this result for the *RASSF1A* CGI promoter and observed that the -400 bp region which harbors the *ZMYND10* TES exhibited increased methylation levels compared to the +400 bp region (Fig. S2). This is consistent with previous findings that CGI shores methylation was associated with gene repression^46–49^.

In addition, the functional annotation of hypermethylated CGI in lung cancer revealed a correlation with bivalent histone H3K27me3 modifications that serve as polycomb target sites (Table S1). It has been suggested that such bivalent chromatin patterns may predispose tumor suppressor genes to epigenetic silencing^12, 50, 51^. Furthermore, we observed a lung cancer-specific increase of the expression of the polycomb repressive factor EZH2 (Fig. S4). *EZH2* overexpression and aberration are frequently observed in cancer, including lung tumors^10, 32, 52^. Notably, *RASSF1A*, as well as *PAX5* and *MEIS2* have also been previously characterized as polycomb target genes^53–55^. In the present study, we found lung cancer specific hypermethylation of *PAX5* and *MEIS2* (Table 1). Aberrant methylation of *PAX5* and *MEIS*, which encode transcription factors harboring a homeobox domain^56, 57^, has already been reported in different cancer entities^16, 17, 20^. In accordance with this, our data also suggest that homeobox domain containing genes are significantly associated with CGI hypermethylation in lung cancer (Fig. S1).

In summary, we identified a novel putative epigenetic regulatory mechanism that is involved in the inactivation of a lung cancer related gene was identified. Our results suggest that the downstream gene promoter could be susceptible to epigenetic silencing when organized in tandem orientation with a CGI harboring a TES of the upstream gene and a TSS of the downstream gene. As the *RASSF1A* tumor suppressor gene represents such a downstream tandem gene, the epigenetic mechanism was confirmed for the *RASSF1A* promoter. HDAC and CPSF1 were identified as factors involved in this repressive effect.

## Materials and Methods

### Cell culture and tumor cells

The A549, H322 and A427 human cell lines (ATCC) were maintained in DMEM F12 medium (Invitrogen) supplemented with 10% heat-inactivated fetal bovine serum (PAA, Austria), 100 units/ml penicillin, 0.1 mg/ml streptomycin, and 2 mM L-glutamine at 37 °C in a 5% CO_2_, 95% O_2_. NHBEC were obtained from Clonetics (Belgium) and grown in BEGM. TREx293 cells, which stably express the Tet repressor (Thermo Fisher, USA), were transfected with the expression vector pcDNA4TO and selected with Zeocin (Invitrogen). HEK293T and TREx293 were transfected using polyethylenimine. All patients gave a written consent at initial clinical investigation. Consent has been obtained to publish in an online-access publication, if the information could lead to the identification of a study participant. The study and experimental protocols were approved by the ethical committee of the Medical Faculty of University Halle-Wittenberg, Germany^2^. All experiments were performed in accordance with relevant guidelines and regulations.

### Infinium HumanMethylation450K BeadChip

For the bead chip array 500 ng of genomic DNA was treated with bisulfite and Infinium Beadchip (Illumina) analysis was performed by Life & Brain GmbH (Bonn). The Illumina HumanMethylation450 K panel targets CpG sites located within the proximal promoter regions of transcription start sites of consensus coding sequences in the NCBI Database (Genome Build 36; www.ncbi.nlm.nih.gov). This bead chip technology allows the assessment of 482,421 highly informative CpG sites per sample at single-nucleotide resolution^58^. Relative methylation levels and differential methylation were calculated using the default settings in GenomeStudio software (Illumina). All subsequent calculations were based on average beta values and DiffScores as extracted from the GenomeStudio analysis. Beadchip methylation data has been deposited at the Gene Expression Omnibus (GEO) repository: GSE92843.

### Methylation analysis

DNA was isolated by phenol-chloroform extraction and then bisulfite treated prior to pyrosequencing^59^. A total of 200 ng was used for PCR with the primers listed in Table S2. Methylation status was quantified utilizing a sequencing primer with PyroMark Q24 utilizing a sequencing primer (Qiagen). M.SssI methylase (NEB) was used for the *in vitro* methylation of genomic DNA.

### Expression analysis

RNA was isolated using the Isol-RNA lysis procedure (5´Prime) from cell lines. RNA was digested with DNase (Fermentas) and then reverse transcribed as previously described^60^. RT-PCR was performed with the primers listed in Table S2. qRT-PCR was performed in triplicates with the SYBR Master Mix (Life Technologies) using a Rotor-Gene 3000 (Qiagen). Expression analysis of 49 primary lung SCCa, 41 lung AdCa, 15 non-malignant lung disease samples and 6 normal lung tissues was performed using a micro array generated and were previously described^2^.

### Cloning of the tandem reporter system

A 2.3 kb fragment of the *ZYMND10*-*RASSF1A* locus was amplified from fibroblast DNA with primers RF1AXHOASEU1: 5′-CTCGAGATTAATACTGTGGAGGGCTGGAAGACCGG and PSL1: 5′-GAATTCACCGGTTCAGGCTCCCCCGACATGGC and cloned into the pRLnull vector (Promega). EGFP was obtained form p*EGFP*-C1 and cloned upstream in the 2.3-kb *RASSF1A* pRLnull construct. The stop codon of *EGFP* was modified by *in vitro* mutagenesis (Primer StopEGFPMutL1: 5′-GACTCAGATCTCGAGATCCTGCCTCTACTCAC and the reverse complement primer) to allow expression of the *EGFP*-*ZYMND10* fusion protein. The EGFP-2.3-RLuc cassette was then cloned into pcDNA4/TO/myc-His-C and the resulting construct was termed TO-EGFP-2,3-RLuc. To generate TO-EGFPpA-2,3-RLuc the 312 bp polyA sequence of SV40 was amplified from the vector pEGFP-C1 (EGFPBsiWIU1: 5′-ACCGTACGCGAGCTCAAGCT and EGFPBsiWIL1: TGCGTACGTAAGATACATTG) and cloned in a novel BsiWI site 3′ of EGFP (generated by *in vitro* mutagenesis with primer BsiwIMutU1: 5′-AGGCAGGATCTCGAGATCTGAGCGTACGCTTGTACAGCTCGTCCATGCCG and the reverse complement). Finally, in the construct TO-EGFP-2,3-RLuc a 1.8 kb XhoI fragment of the ZYMND10 locus was deleted and the backbone was re-ligated to generate the TO-EGFP-0,5-RLuc construct.

### Constructs

The following vectors and plasmids were used: pEGFP-C1 (Clontech); pcDNA4/TO/myc-His-C (Invitrogen, USA); pCMV-Tag1 (Stratagene), pRL-Null (Promega) and pGL3.1 (Promega). The cDNA of CPSF1 was obtained in pOTB7 (IRAUp969E0360D; Source BioScience, UK). By *in vitro* mutagenesis a BamHI (BamEcoMutU1: 5′-GTCGGCTCCAACTGCCAGGATCCGAATTCGCCCGGGTT and reverse complement primer) and EcoRV (EcoRVMutU1: 5′-CGTCACCGCCCACTTCTAGATATCTGGATGCCGTCACCACCAG and the reverse complement primer) restriction sites were generated flanking the cDNA and the ORF of CPSF1 was cloned in frame in pCMV-Tag1. HDAC1, HDAC2, HDAC3-FLAG, HDAC4-FLAG, HDAC5-FLAG, HDAC6, HDAC8, HDAC10-FLAG and Myc-Sirtuin 1 were obtained from Lienhard Schmitz (Justus-Liebig-University, Germany). The constructs were confirmed through conventional sequencing.

### Luciferase assays

Luciferase promoter assays were performed using the Dual-Luciferase Reporter System (Promega) and assessed using the microplatte illuminometer ORIONL (Berthold). The transfection efficiency was controlled using the corresponding empty control vector (pGL3.1: 300 ng firefly luciferase respectively). The obtained data were normalized to the corresponding control vectors.

### Western blot and antibodies

For western blot analysis, 20–30 µg protein lysates were separated using 12% PAGE-SDS gels and blotted on a PVDF membrane (Amersham). All antibodies were obtained from Santa Cruz Biotechnologies (Dallas, USA): anti GAPDH FL-335 (1:10000), anti Myc-Tag (1:2000) and anti GFP rabbit polyclonal serum (1:1000). For detection a goat HRP-coupled anti rabbit antibody (sc-2004) was utilized and detected with an enhanced chemiluminescence reagent (WCI-HRP-Substrate, Millipore) using a VersaDoc Imager.

### Chromatin immunoprecipitation (ChIP) and antibodies

ChIPs was performed as described previously^13^. DNA was recovered by using QIAquick PCR Purification Kit (Qiagen) and PCR amplification with the following primers: EGFPRTF1: 5′-ACGTAAACGGCCACAAGTTC, EGFPRTR1: 5′-AAGTCGTGCTGCTTCATGTG, RF1ALucChIPU1: 5′-GCTCTCCTCAGCTCCTTCC, RF1ALucChIPL1: 5′-GTGCCTCACGACCAACTTCT. The TO/EGFP and RASSF1A promoters were amplified using the primers F1/R1 (187 bp), and U1/L1 (195 bp) respectively. qPCR was performed in triplicate using the SYBR Select Master Mix (Life Technologies) using Rotor-Gene 3000 (Qiagen).

### Statistical analysis

Statistical and correlations analyses were performed using R version 3.1.3 (R Foundation). The data are presented as the means of biological triplicates ±S.D. The p-values were quantified by Student’s unpaired t-test, by Chi square test or by Wilcoxon rank-sum test. The differences are considered significant if: *p < 0.05; **p < 0.01; ***p < 0.001.

## Electronic supplementary material

Supplementary Information
