# Supplementary material for: Epigenetic silencing of downstream genes mediated by tandem orientation in lung cancer
Source: Sci Rep. 2017 Jun 20;7:3896. doi: 10.1038/s41598-017-04248-w (PMC5478622; doi:10.1038/s41598-017-04248-w)

# Supplementary Information

# Epigenetic silencing of downstream genes mediated by tandem orientation in lung cancer

**Steffen Kiehl[1], Tobias Zimmermann[1], Rajkumar Savai[2,3], Soni S. Pullamsetti[2,3], Werner Seeger[2,3], Marek Bartkuhn[1], Reinhard H. Dammann[1,3,*]**

[1]Institute for Genetics; Justus-Liebig-University, 35392 Giessen, Germany

[2]Department of Lung Development and Remodeling, Max-Planck-Institute for Heart and Lung Research, 61231 Bad Nauheim, Germany

[3]German Center for Lung Research (DZL), Universities of Giessen and Marburg Lung Center, 35392 Giessen, Germany

***Correspondence:** Reinhard.Dammann@gen.bio.uni-giessen.de

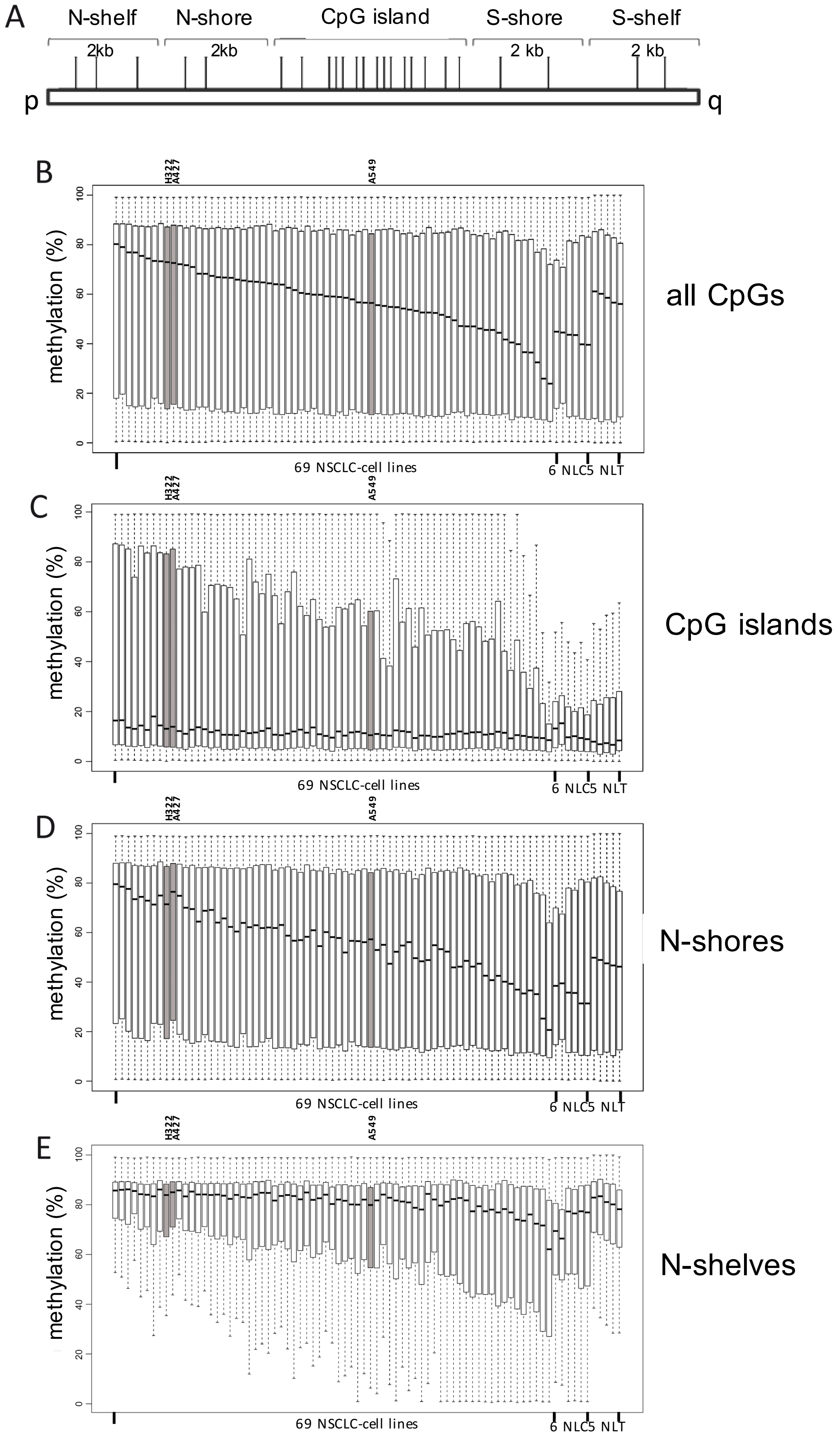


**Fig S1. Increased methylation of CpG island-associated regions (CGIR) occurs in lung cancer cell lines. A.** GC and CpG rich genomic elements, termed CpG islands are flanked by 2 kb shore and 2 kb shelf regions. Depending on their chromosome orientation from the p to q arm, these regions are denoted as N or S-shores and shelves. **B.** CpG methylation of 69 NSCLC-cell lines, six normal lung cell lines (NLC) and five normal lung tissues (NLT) were obtained from NLCBI-GEO-Accession: GSE36216 and GSE52401 [21,22]. Methylation levels were analyzed by box plots are shown. Samples are sorted depending on their median methylation and origin (see above). H322, A427 and A549 are highlighted in gray. Methylation of CpG island-associated CpGs (n=150,254). (**C**), N-shore-associated CpGs (n = 62,870) (**D**), and N-shelf-associated CpGs (n = 24,844) (**E**) in 69 NSCLC, 6 NLC, and 5 NLT samples is plotted.

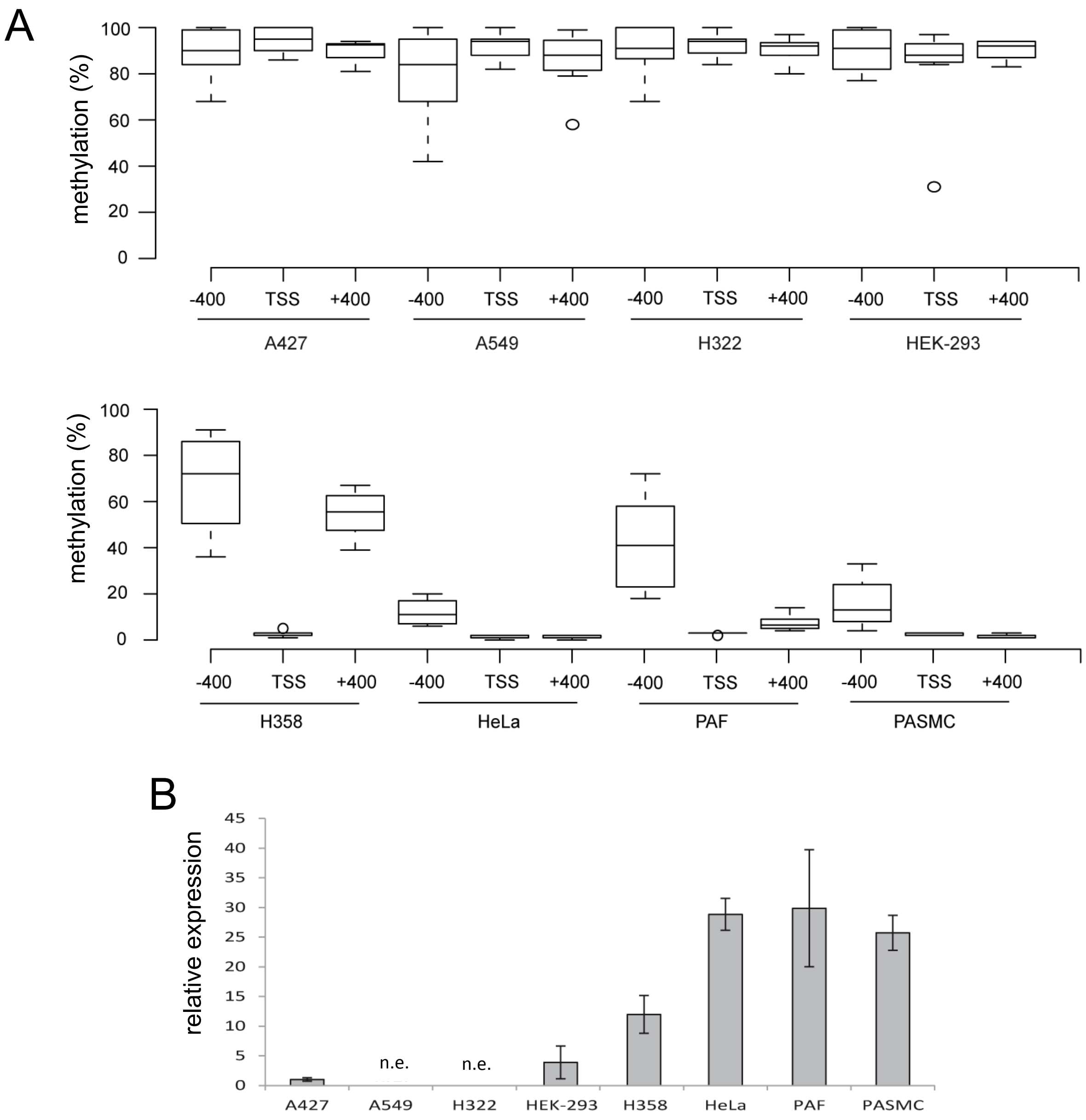


**Fig S2. Methylation and expression of the *RASSF1A* tumor suppressor gene. A.** Methylation levels of the transcriptional start site (TSS) of *RASSF1A* and the flanking upstream (-400) and downstream (+400) regions in lung cancer cell lines (A427, A549, H322 and H358), HEK-293, HeLa, pulmonary arterial fibroblasts (PAF) and pulmonary arterial smooth muscle cells (PAMSC). **B.** Expression of *RASSF1A* as measured by qRT-PCR. Expression of *RASSF1A* was quantified by real time RT-PCR and normalized to actin levels (A427=1; n.e.= no expression).

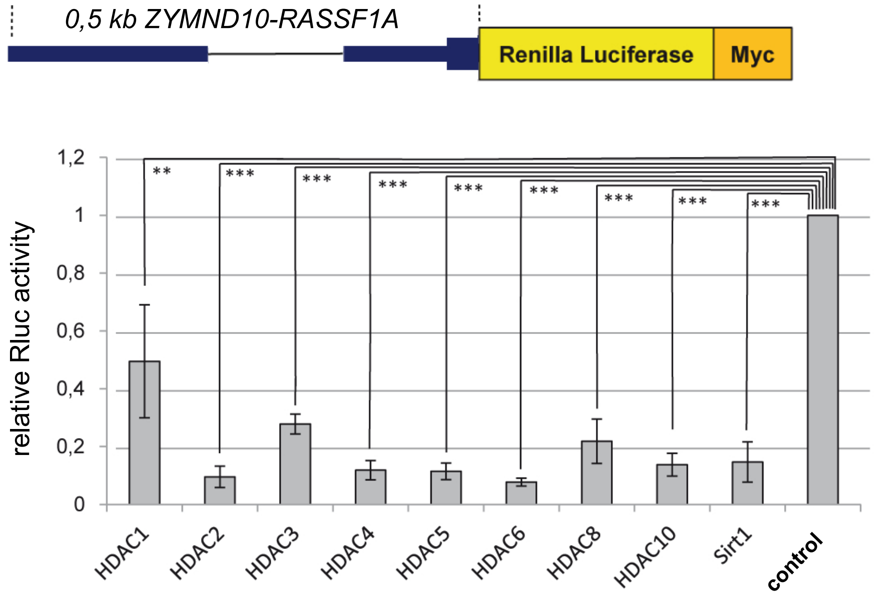


**Fig. S3. Expression of histone deacetylases (HDAC) represses the *RASSF1A* promoter.** The 500 bp *RASSF1A* promoter including the 5' UTR and 17 bp of Exon1a was ligated in-frame to a Myc tagged *Renilla* luciferase (RLuc). Black boxes represent exons. The 0.5 kb *RASSF1A* promoter construct was stably transfected into HEK293 cells. RLuc activity after 3 days of transfection with the indicated histone deacetylases (HDAC), Sirt1, or the control vector and normalized to transiently transfected firefly luciferase and the control (=1) is shown. **p<0.01 and ***p<0.001.

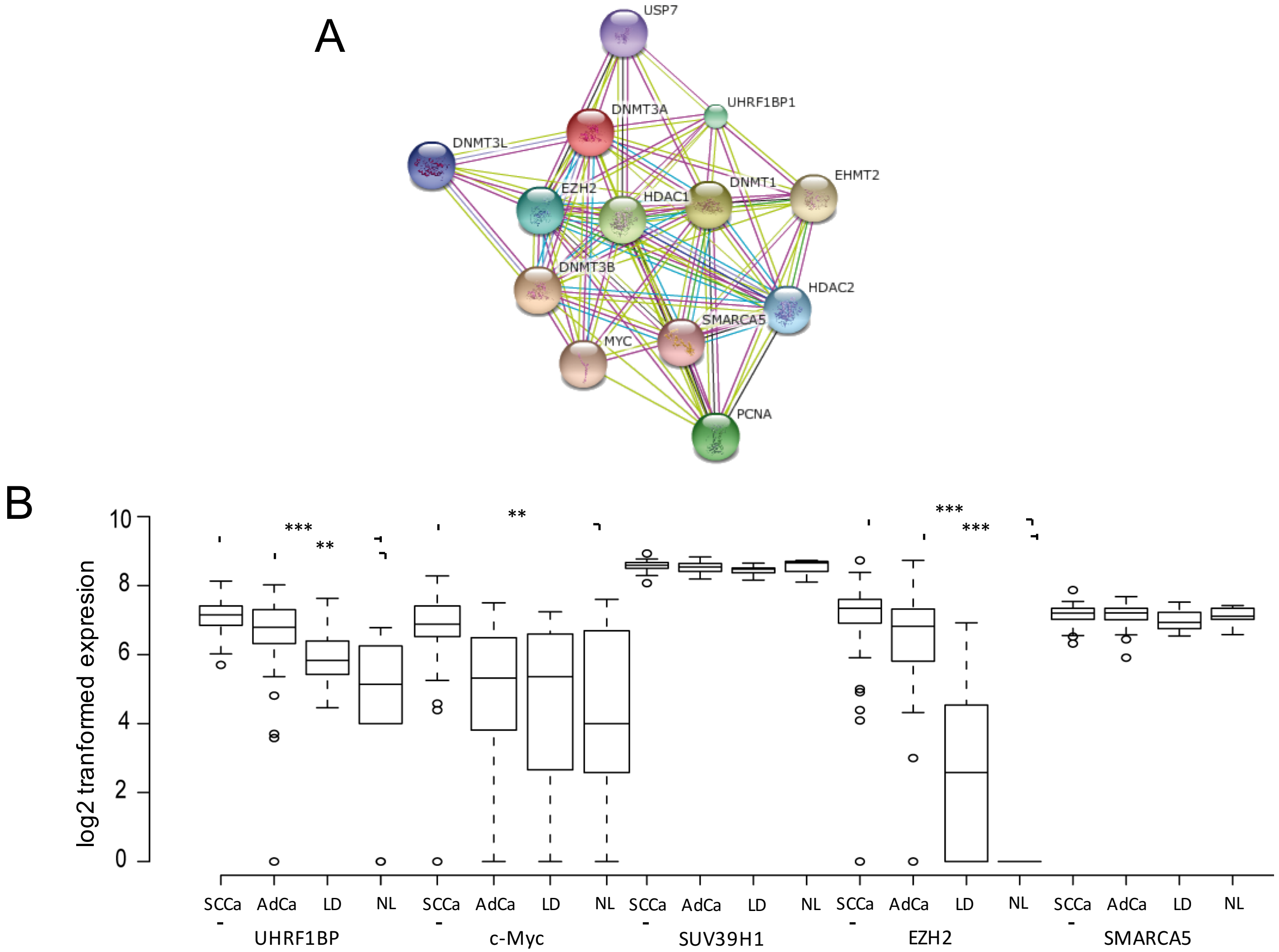


**Fig. S4. Expression analysis of additional DNMT interaction partners in lung samples. A.** Schematic overview of direct interaction partners of *DNMT1*, *DNMT3A*, *DNMT3B* according to the string database (http://string-db.org). **B.** Expression of *UHRF1*, *c-MYC*, *SUV39H1*, *EZH2* and *SMARCA5* as determined by microarray analysis in 49 squamous cell cancer (SCCa), 41 adenocarcinoma (AdCa), 15 non malignant lung disease samples (LD) and 6 normal lung tissues (NL) [2]. Data are depicted as log2 transformed expression. **p<0.01 and ***p<0.001.

**Table S1: Overview on the GO Terms of hypermethylated CGIR in lung cancer cell lines.** GO-Terms with most significant enrichment by q-value (false discovery rate/FDR adjusted p-value) are shown based on the indicated ontology data bank.

| Ontology (data bank) | Classification | GO-term name (standard name) | Hyper FDR q-value | Number of genes vs. total number of annotated genes |
|---|---|---|---|---|
| GO Consortium | molecular function | sequence-specific DNA binding (ID: GO:0043565) | $2.5680 \times 10^{-113}$ | 333 of 697 |
| InterPro | analysis by classifying into families and predicting domains and important sites. | homeobox domain (ID: IPR001356) | $4.6471 \times 10^{-127}$ | 162 of 243 |
| MSigDB *Perturbation* | gene expression profiles of genetic associated disorders | H3K27me3 bound genes (ID: BENPORATH_ES_WITH_H3K27ME3) | $3.1532 \times 10^{-159}$ | 520 of 1059 |
| | | Polycomb protein EED target (ID: BENPORATH_EED_TARGETS) | $2.2925 \times 10^{-147}$ | 454 of 1005 |
| | | Polycomb Repression Complex 2 targets (ID: BENPORATH_PRC2_TARGETS) | $3.6522 \times 10^{-130}$ | 324 of 613 |
| MSigDB *Pathway* | gene sets from the pathway databases | regulation of beta-cell development (ID:REACTOME_REGULATION_OF_BETA_CELL_DEVELOPMENT) | $1.8217 \times 10^{-10}$ | 16 of 30 |
| | | Maturity onset diabetes of the young (ID:KEGG_MATURITY_ONSET_DIABETES_OF_THE_YOUNG) | $3.5505 \times 10^{-10}$ | 14 of 25 |
| MSigDB *Predicted promoter motifs* | Gene sets that share a cis-regulatory motif and transcription factor binding site | Motif VSX1: visual system homeobox 1 homolog, CHX10-like (zebrafish) (ID: V$CHX10_01) | $3.5309 \times 10^{-34}$ | 78 of 211 |
| | | Motif PAX4: paired box gene 4 (ID: V$PAX4_02) | $3.4385 \times 10^{-31}$ | 74 of 225 |
| | | Motif CRX: cone-rod homeobox (ID: V$CRX_Q4) | $2.4854 \times 10^{-28}$ | 102 of 277 |
| | | Motif POU3F1: POU domain, class 3, transcription factor 1 (ID: V$TST1_01) | $6.8428 \times 10^{-27}$ | 87 of 246 |
| | | Motif GFI1: growth factor independent 1 (ID: TGATTTRY_V$GFI1_01) | $2.1314 \times 10^{-26}$ | 85 of 278 |
| | | Motif POU2F1: POU domain, class 2, transcription factor 1 (ID: V$OCT1_04) | $2.2480 \times 10^{-25}$ | 78 of 227 |

**Supplement Table 2.** List of primers

| Primer | Sequence (5'-3') | Use |
|---|---|---|
| UHE2ab | GGCTGGGAACCCGCGGTG | RASSF1A expression |
| L27111 | TCCTGCAAGGAGGGTGGCTTCT | RASSF1A expression |
| ßACTF | CCTTCCTTCCTGGGCATGGAGTC | ACTB |
| ßACTR | CGGAGTACTTGCGCTCAGGAGGA | ACTB |
| RF1APYROFOR | AGTTTGGATTTTGGGGGAGG | RASSF1A TSS methylation |
| RF1APYROBIO | Biotin-CAACTCAATAAACTCAAACTCCCC | RASSF1A TSS methylation |
| RF1A-500BSU1 | GGTGATAGAGTTAAATGAGGGTTGTA | RASSF1A -400 methylation |
| RF1A-300BSL1 | Biotin-AACACACTTAACCTACCCACTAAAT | RASSF1A -400 methylation |
| +400/+600BSU1 | GGAAGGGGTAGTTAAGGG | RASSF1A +400 methylation |
| +400/+600BSL1 | Biotin AAACAACCACCTCTACTCAT | RASSF1A +400 methylation |
| RF1APYROSEQ | GGGGTTAGTTTTGTGGTTT | RASSF1A TSS methylation sequencing |
| RF1A-500BSSeq | AGAATGTATTTTTGAATTTTAAGA | RASSF1A -400 methylation sequencing |
| 400BSSeqU3 | GGGGTAGTTAAGGGG | RASSF1A +400 methylation sequencing |

**Supplementary information of Figure 5:**

Analysis of different tandem constructs by western blot. Two TREx clones (B7 and A7) of the stable transfected TO-EGFP-2,3-RLuc were induced with 4 mM Dox (+) for four days and the protein levels were subsequently analyzed by western blot. Protein lysates were separated on SDS PAGE and blotted. For detection of the indicated proteins primary antibody against myc, EGFP and GAPDH were utilized. Additionally the expression of *RLuc* and *EGFP* were analyzed in two TO-EGFPpA-2,3-RLuc TRex clones (A1 and A2) and the C2 clone of TO-EGFP-0,5-RLuc by western blot:

**12 Full-length western blots of Figure 5 are included:**

1. TREx A1 and TREx A2 probed for EGFP-ZMYND10 expression
2. TREx A1 and TREx A2 probed for GAPDH expression
3. TREx A1 and TREx A2 probed for RLuc-Myc expression
4. TREx A7 probed for EGFP-ZMYND10 expression
5. TREx A7 probed for GAPDH expression
6. TREx A7 probed for RLuc-Myc expression
7. TREx B7 probed for EGFP-ZMYND10 expression
8. TREx B7 probed for GAPDH expression
9. TREx B7 probed for RLuc-Myc expression
10. TREx C2 probed for EGFP-ZMYND10 expression
11. TREx C2 probed GAPDH expression
12. TREx C2 probed RLuc-Myc expression

**Details of western blot and antibodies**

For western blot analysis, 20-30 µg protein lysates were separated using 12% PAGE-SDS gels and blotted on a PVDF membrane (Amersham). All antibodies were obtained from Santa Cruz Biotechnologies (Dallas, USA): anti GAPDH FL-335 (1:10000), anti Myc-Tag (1:2000) and anti EGFP rabbit polyclonal serum (1:1000). For detection a goat HRP-coupled anti rabbit antibody (sc-2004) was utilized and detected with an enhanced chemiluminescence reagent (WCI-HRP-Substrate, Millipore) using a VersaDoc Imager.

1. TREx A1 and TREx A2 probed for EGFP-ZMYND10 expression

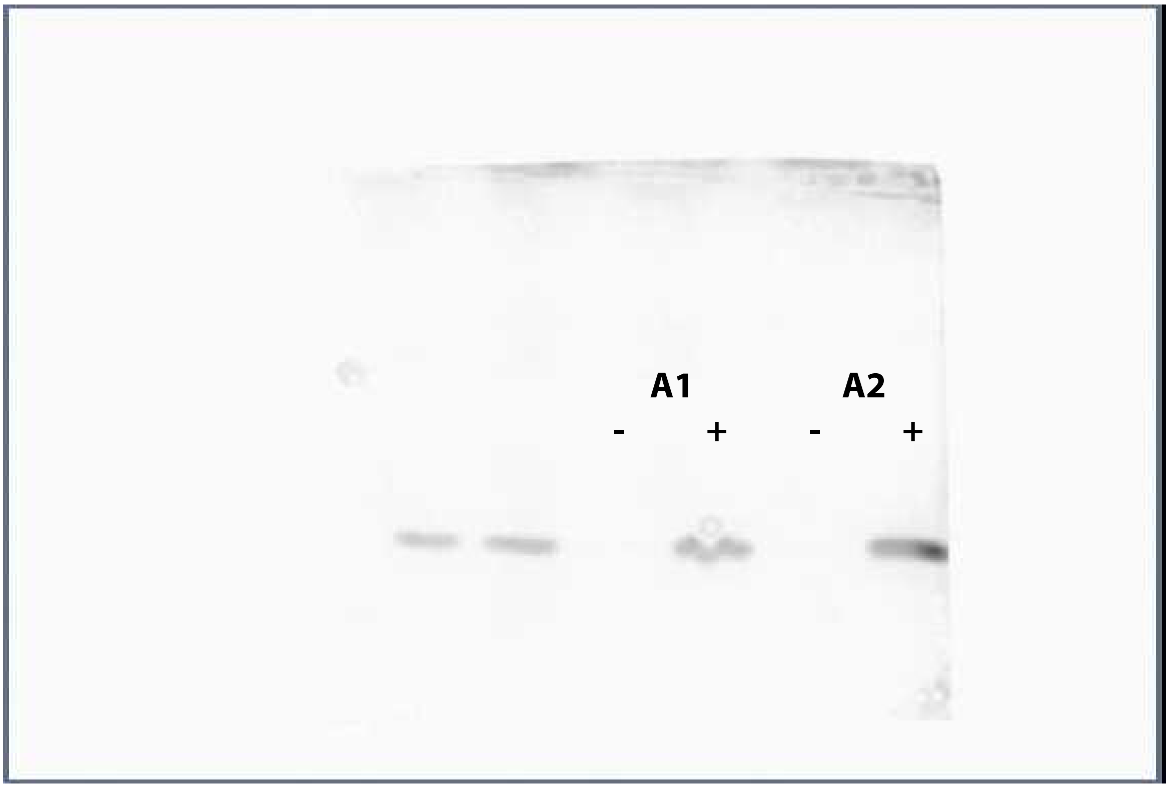

**2. TREx A1 and TREx A2 probed for GAPDH expression**

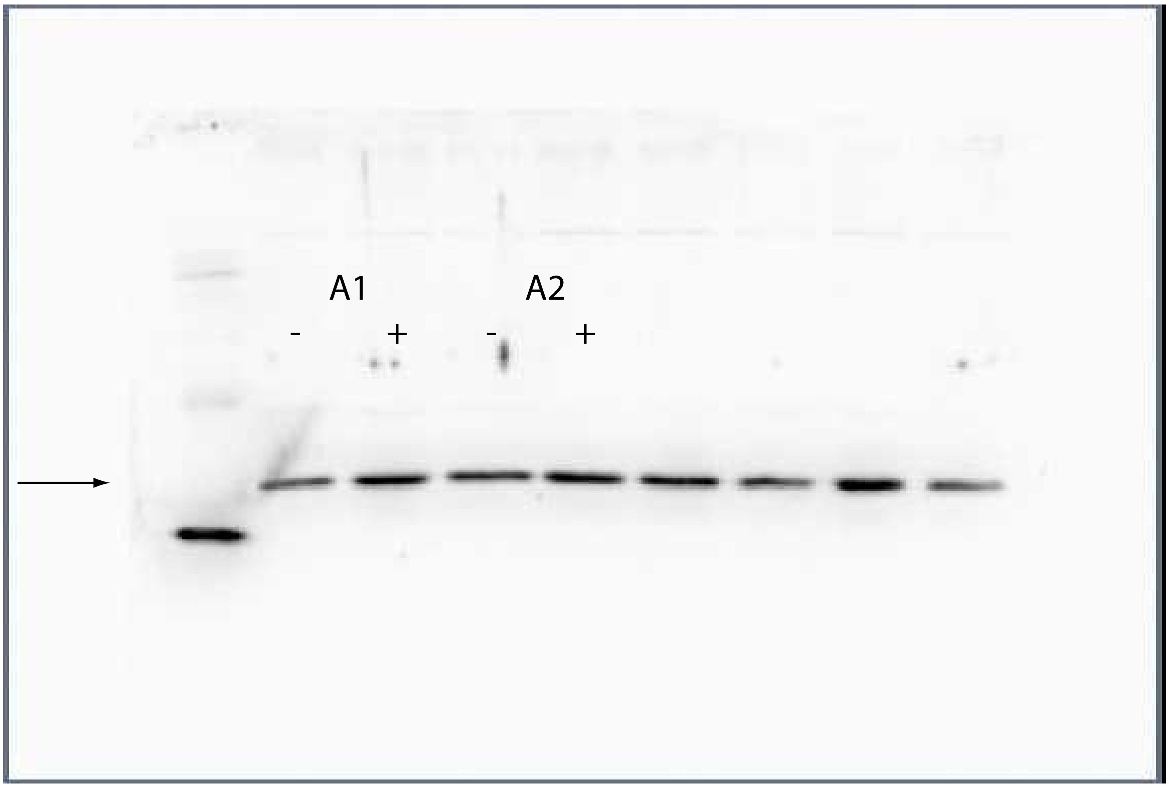

3. TREx A1 and TREx A2 probed for RLuc-Myc expression

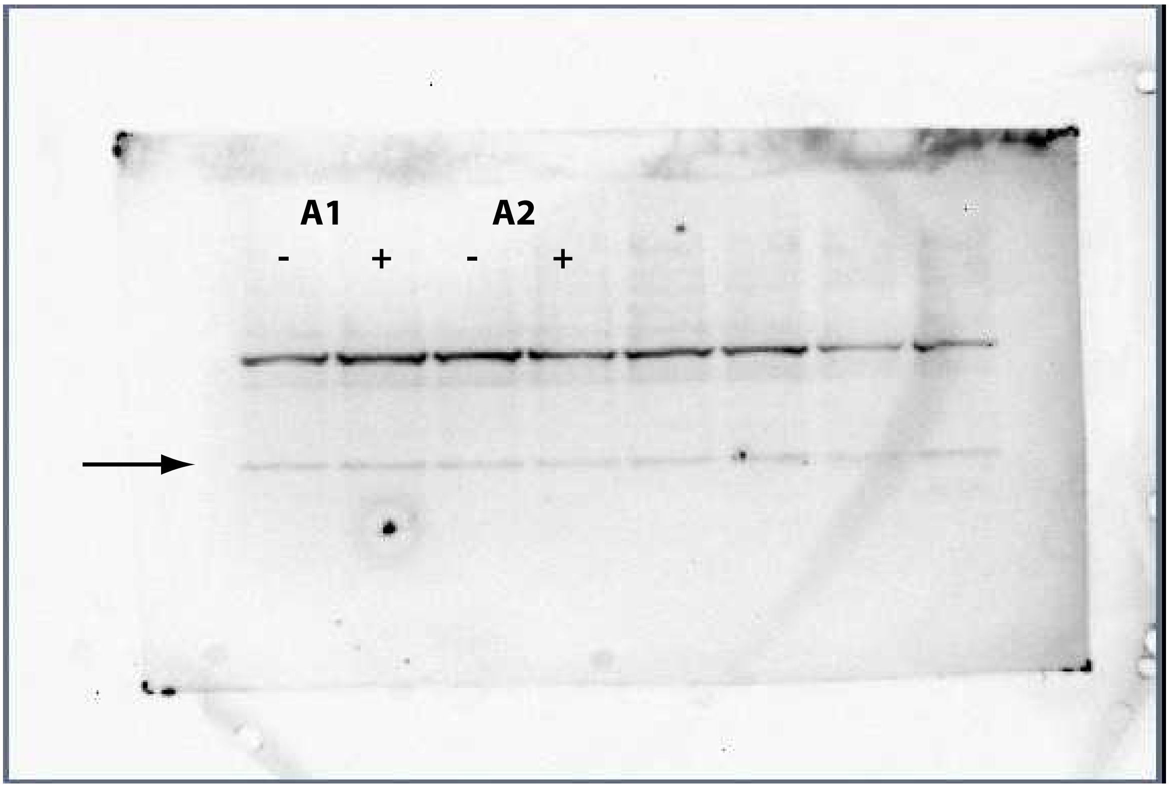

**4. TREx A7 probed for EGFP-ZMYND10 expression**

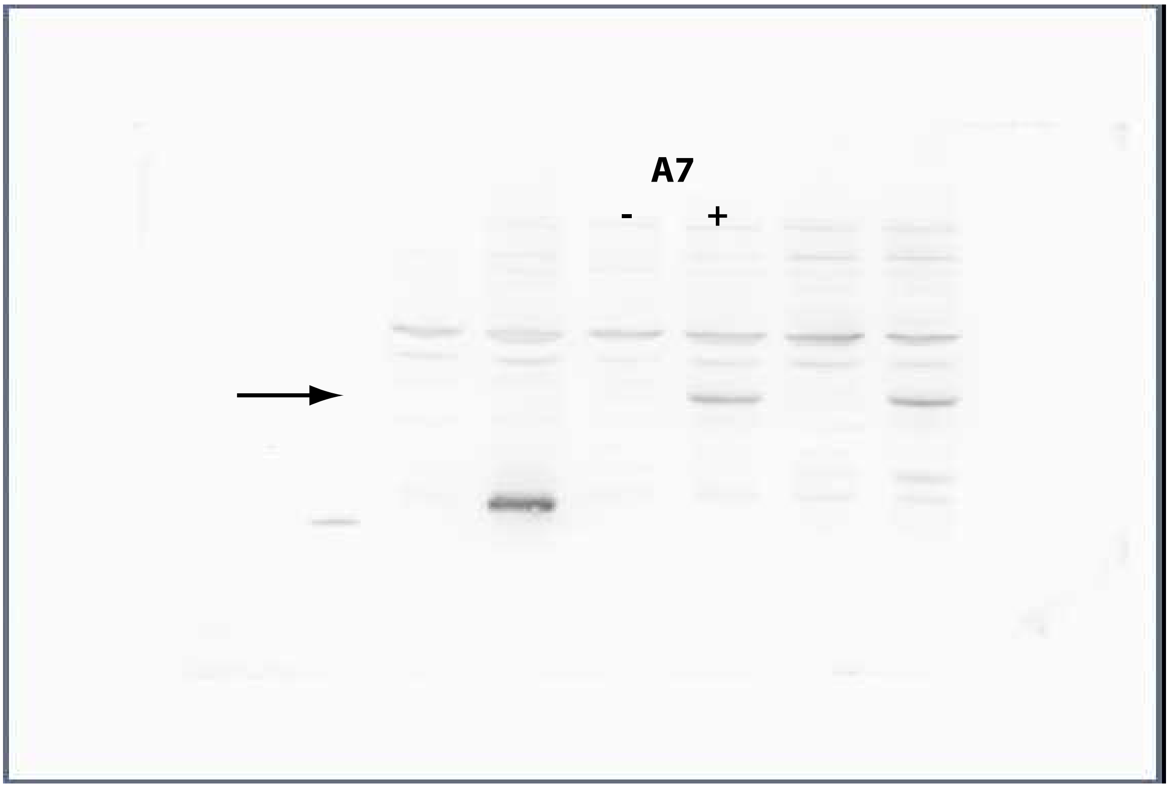

**5. TREx A7 probed for GAPDH expression**

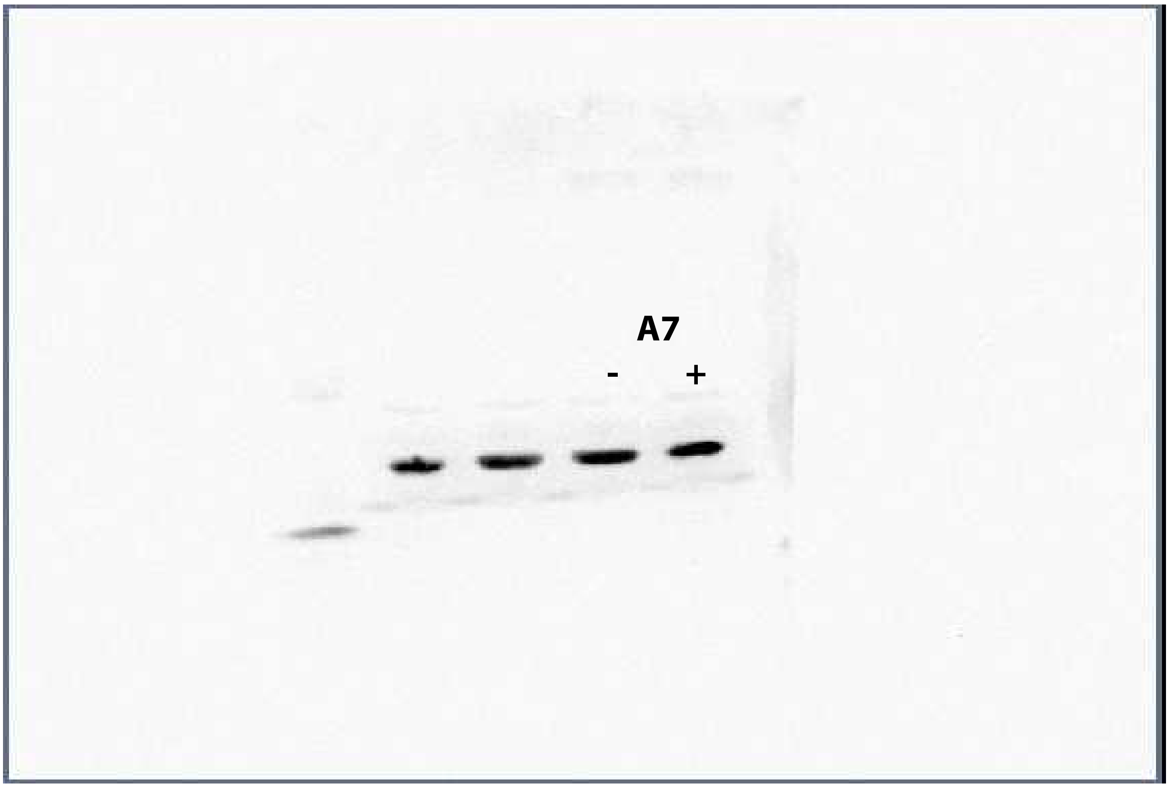

6. TREx A7 probed for RLuc-Myc expression

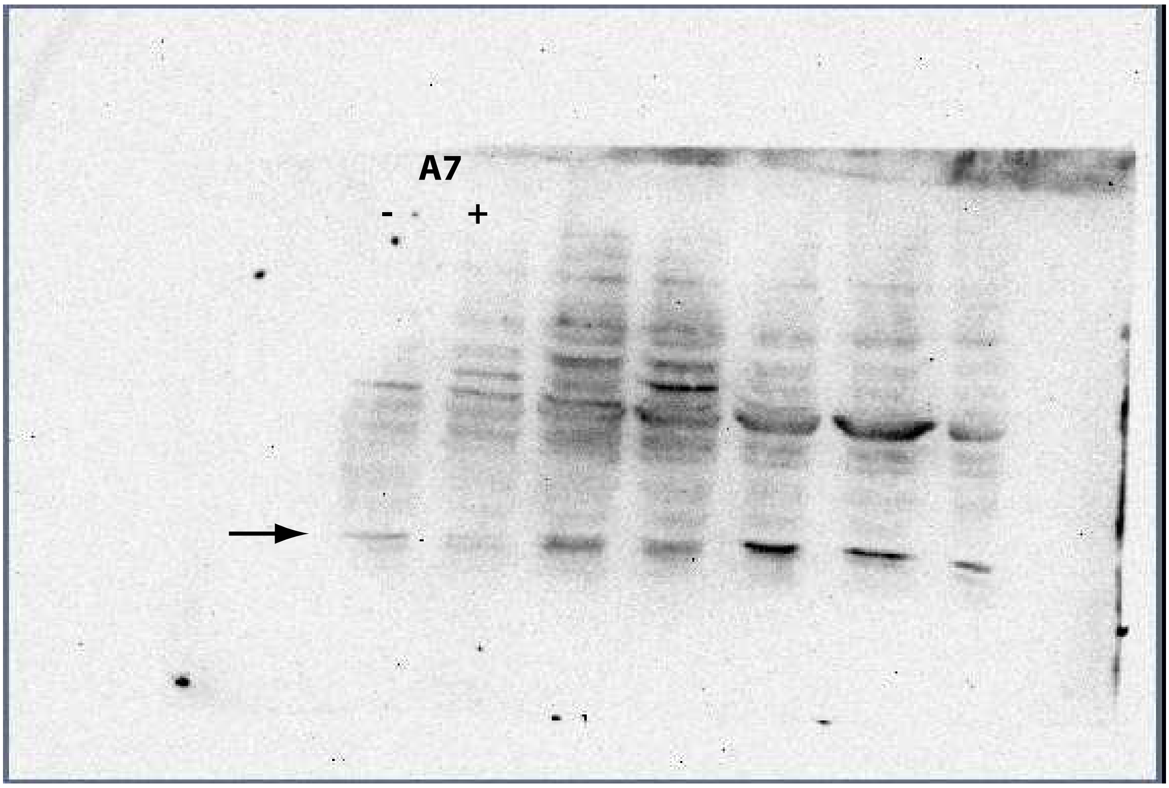

7. TREx B7 probed for EGFP-ZMYND10 expression

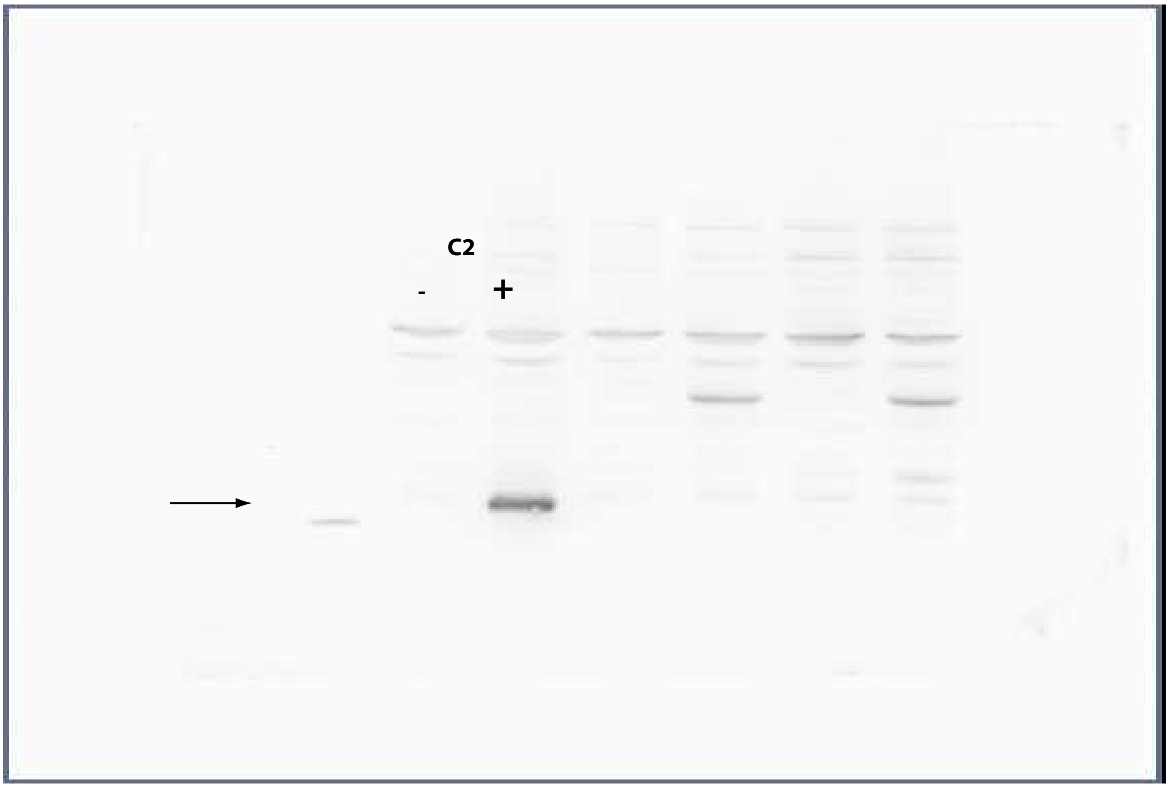

**8. TREx B7 probed for GAPDH expression**

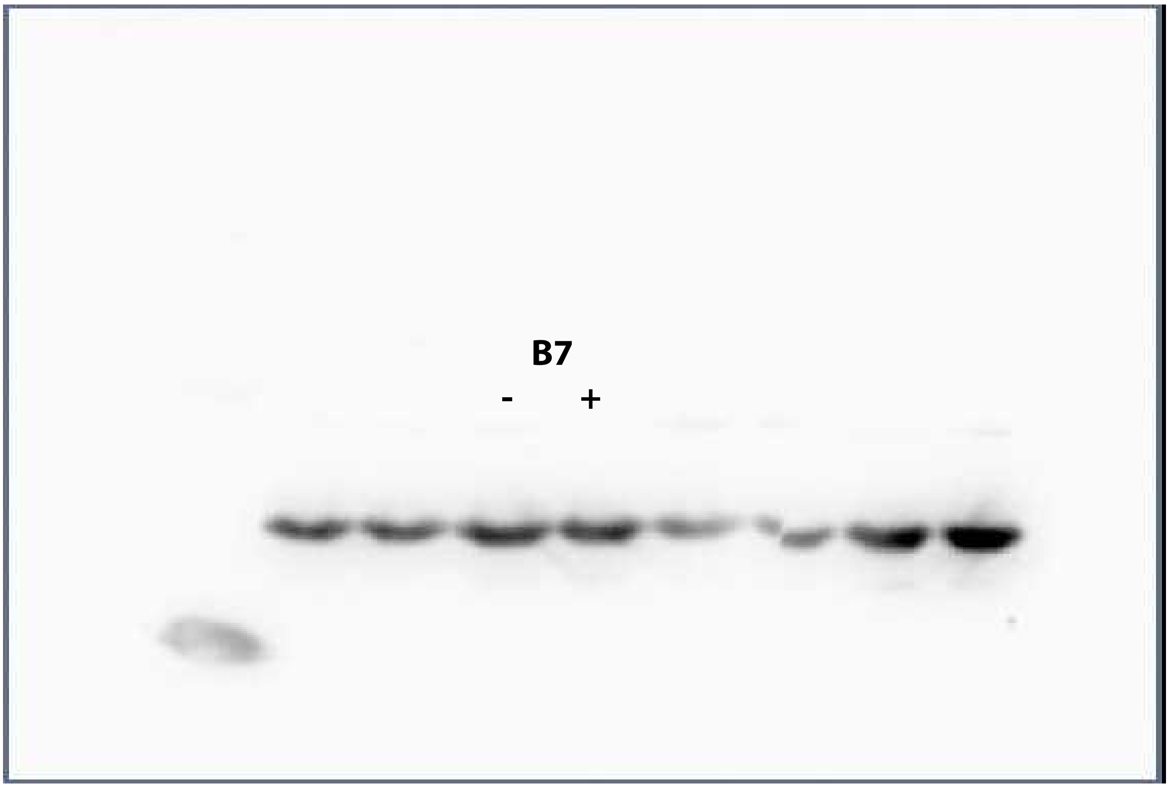

**9. TREx B7 probed for RLuc-Myc expression**

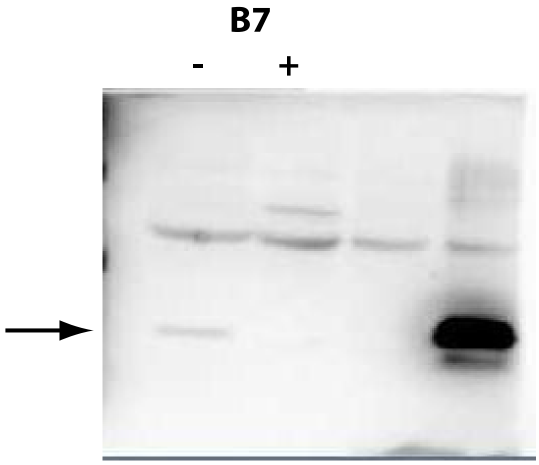

**10. TREx C2 probed for EGFP-ZMYND10 expression**

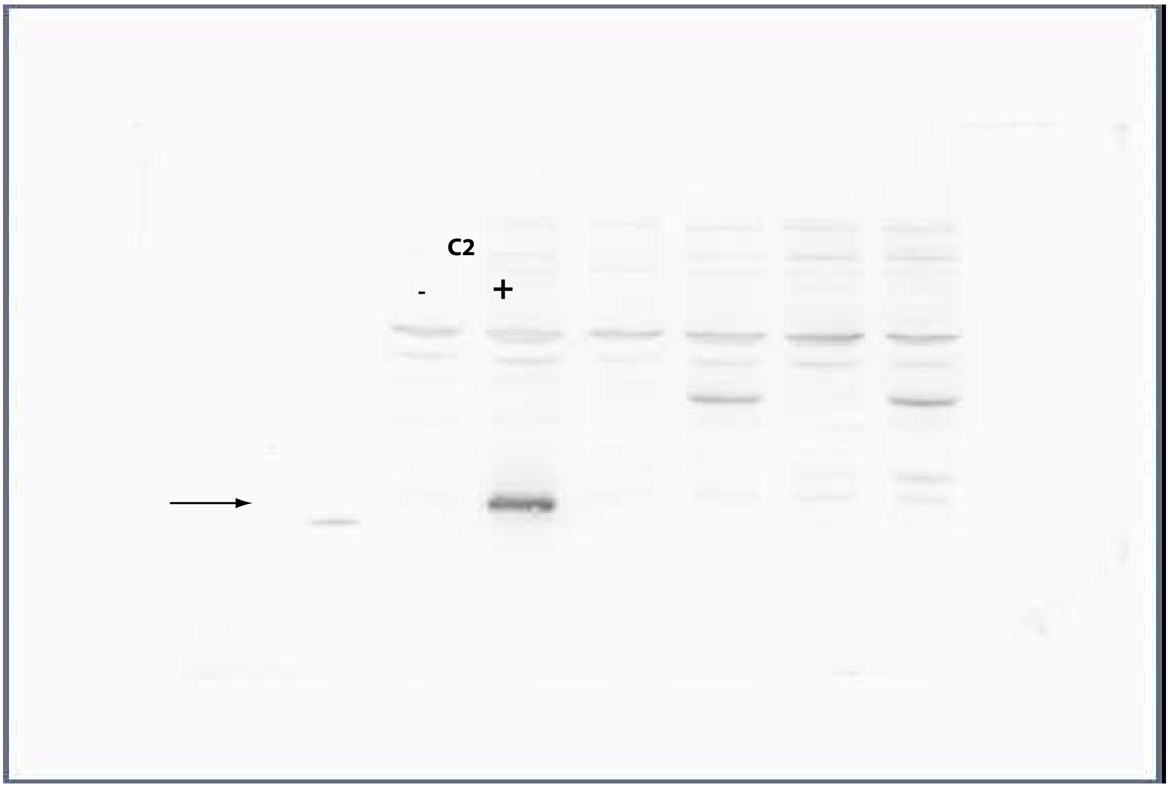

**11. TREx C2 probed GAPDH expression**

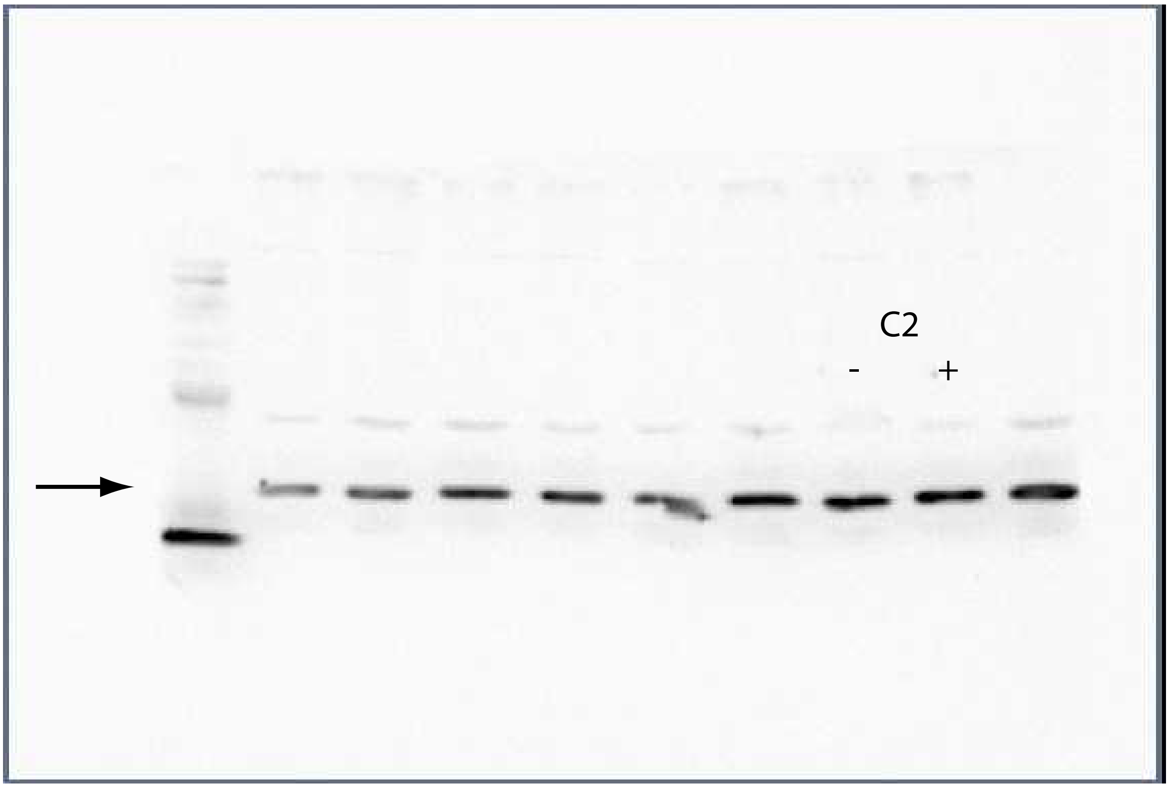

12. TREx C2 probed RLuc-Myc expression

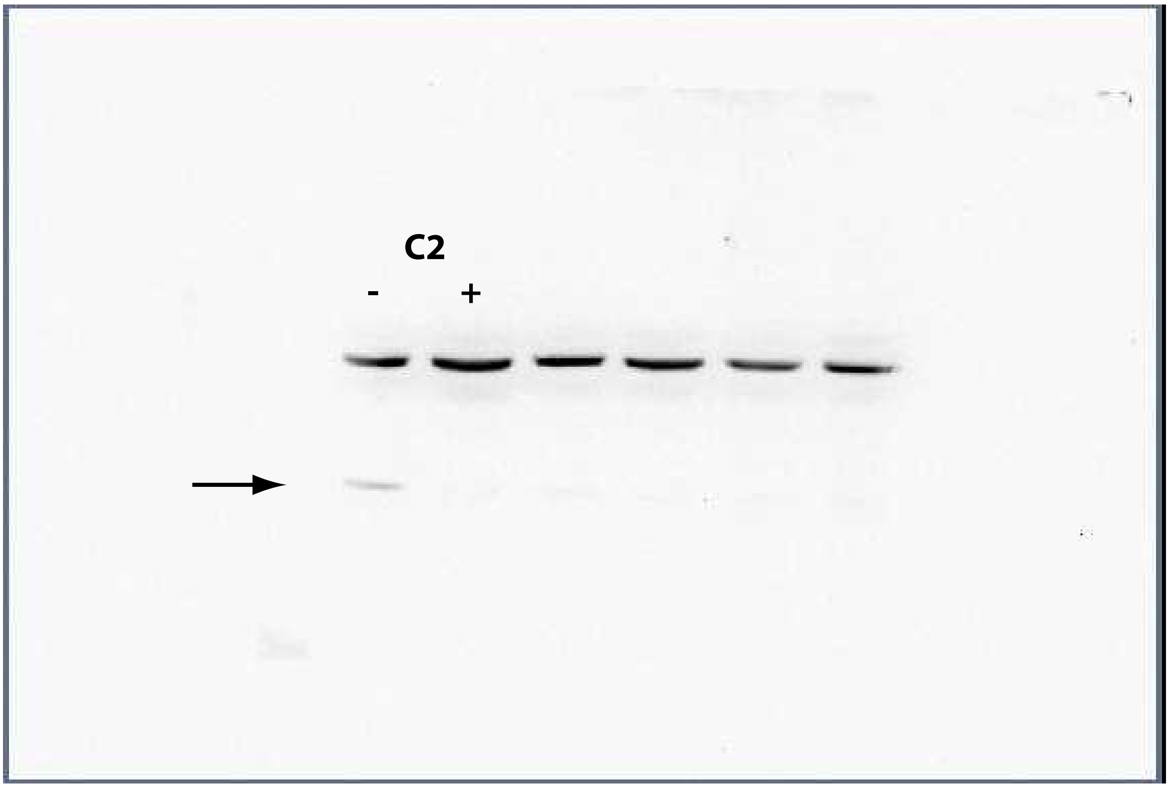

Supplement: Supplementary file 1 — Supplementary Information [file 41598_2017_4248_MOESM1_ESM.pdf]
